# In-Depth Analysis of the Effect of Fragmentation on the Crystallization-Driven Self-Assembly Growth Kinetics of 1D Micelles Studied by Seed Trapping

**DOI:** 10.3390/polym13183122

**Published:** 2021-09-16

**Authors:** Gerald Guerin, Paul A. Rupar, Mitchell A. Winnik

**Affiliations:** 1 Shanghai Key Laboratory of Advanced Polymeric Materials, Key Laboratory for Ultrafine Materials of Ministry of Education, School of Materials Science and Engineering, East China University of Science and Technology, Shanghai 200237, China; 2 Department of Chemistry, University of Toronto, 80 St. George Street, Toronto, ON M5S 3H6, Canada; 3 Department of Chemistry, University of Alabama, Tuscaloosa, AL 35487, USA; parupar@ua.edu; 4 Department of Chemical Engineering and Applied Chemistry, University of Toronto, Toronto, ON M5S 3E2, Canada

**Keywords:** block copolymers, crystallization-driven self-assembly, kinetics, fragmentation, growth

## Abstract

Studying the growth of 1D structures formed by the self-assembly of crystalline-coil block copolymers in solution at elevated temperatures is a challenging task. Like most 1D fibril structures, they fragment and dissolve when the solution is heated, creating a mixture of surviving crystallites and free polymer chains. However, unlike protein fibrils, no new nuclei are formed upon cooling and only the surviving crystallites regrow. Here, we report how trapping these crystallites at elevated temperatures allowed us to study their growth kinetics at different annealing times and for different amounts of unimer added. We developed a model describing the growth kinetics of these crystallites that accounts for fragmentation accompanying the 1D growth process. We show that the growth kinetics follow a stretched exponential law that may be due to polymer fractionation. In addition, by evaluating the micelle growth rate as a function of the concentration of unimer present in solution, we could conclude that the micelle growth occurred in the mononucleation regime.

## 1. Introduction

“Seeing is believing” is a well-known idiom that has found an echo in science, where many breakthroughs involved microscopy techniques. In polymer science, one of the major discoveries was made in 1957 by Andrew Keller [1], while studying homopolymer single crystals by transmission electron microscopy (TEM). Keller confirmed that the thin lamellae of these crystals consisted of folded chains, as first suggested by Storks [2], and then showed that the lamellar thickness of a homopolymer single crystal depends directly on the temperature at which it was grown [3]. Since that time, microscopy techniques have become an important tool to further investigate crucial aspects of polymer crystallization, e.g., crystal morphology [4,5,6,7] and crystal growth kinetics [8,9,10,11].

Several groups have examined the self-assembly in solution of crystalline-coil block copolymers (BCPs) that crystallize to form 1-dimensional (1D) micelles [12,13,14,15,16,17,18]. In several cases, the length and the composition of these micelles can be controlled precisely by adding free block copolymer (“unimer”) with the same crystalline block, leading to the formation of elongated micelles or more complex structures referred to as block co-micelles [19,20,21]. Among these BCPs, those with a polyferrocenyldimethylsilane (PFS) core-forming block have been the most intensely studied. PFS BCPs have allowed the formation of the most advanced structures via stepwise hierarchical assembly [22,23,24,25,26,27,28]. Such sophisticated structures are, however, difficult to create with other crystalline-coil BCPs, a situation compounded by our limited fundamental understanding of the crystallization and growth of these 1D micelles.

To follow the growth kinetics of homopolymer single crystals in solution by microscopy, the crystals need to be isolated from their supersaturated unimer solution at different annealing times. For 2D single crystals, one can sediment the crystals [29] or transfer a crystal suspension quickly from one thermostated bath to another to surround the original growth front with a crystalline layer of different thickness [8]. For 1D core crystalline micelles, Boott et al. [11] followed the growth kinetic of PFS-*b*-PDMS (where PDMS stands for polydimethylsiloxane) core-crystalline micelles by depositing a drop of the micelle solution onto a TEM grid at a given annealing time and measuring the micelle lengths. They evaluated the effect of solvent, unimer concentration and block ratio on the micelle growth rate of PFS-*b*-PDMS micelles at temperatures where micelle fragmentation and dissolution could be ignored.

Furthermore, valuable information about the formation and growth of core-crystalline micelles can be obtained by studying their growth kinetics as a function of polymer concentration. Indeed, crystal growth in solution is considered to be interface-controlled, and the growth-determining step is the attachment of straight-chain segments (“stems”) to the growth front and their rearrangement to form a surface nucleus [30,31,32,33]. Modern theory identifies different growth regimes depending on the number and rate at which stems spread on the crystal face [29,34]. These regimes are predicted to exhibit different dependences on polymer concentration, *c*, and can be identified experimentally by studying the polymer concentration dependence of crystal growth rates, *G*. This relationship is given by *G* ∝ *c^γ^*, where *γ* is the concentration exponent. For polymer single crystals, *γ* ranges from 0.2 to 2 [8,35]. A value of *γ* lower than 1 implies the presence of a barrier to chain deposition at the crystal growth front, while *γ* larger than 1 suggests cooperation between several unimers in solution to form a stable nucleus that would grow on the crystal face.

More recently, we have been particularly interested in understanding the main factors that affect micelles dissolution [36,37]. We noticed that when a seed solution was heated at a temperature where most of the seed dissolved, the surviving seeds could broaden and extend [38]. To further investigate this phenomenon, here we report the growth kinetics study of core-crystalline micelles in the presence of different amounts of unimer, at a temperature where the micelles could both fragment and dissolve. For this purpose, we pre-heated solutions of PFS_53_-*b*-PI_637_ (where PI stands for polyisoprene, and the subscripts represent the degree of polymerization of each block) crystallites at 75 °C, and added different amounts of unimer of the same BCP. After different annealing times, we injected a large excess of PFS_60_-*b*-PDMS_660_ to trap the growing crystallites and measured their length for each annealing time, following a seed-trapping approach developed previously [36]. In-depth analysis of the lengths of the trapped seeds and that of the control samples (without trapping the seeds) allowed us to develop a kinetic model that accounts for the fragmentation of the seeds during their growth. We also showed that the growth kinetics could be well described by a stretched exponential, in agreement with the kinetics study of Boott et al. [11]. To explain these results, we hypothesize that the stretched exponential is caused by polymer fractionation. Finally, from the concentration dependency of the seed growth rates, we showed that the 1D growth occurs via the successive addition of polymer chains to the exposed crystal faces.

## 2. Materials and Method

Decane (99+%) and Karstedt’s catalyst were purchased from Sigma-Aldrich (Oakville, ON, Canada) and used without further purification.

The PFS_53_-*b*-PI_637_ (M_n_, GPC = 56,300, Đ = 1.01) and PFS_60_-*b*-PDMS_660_ were synthesized by one of us and have been reported in ref [39].

### 2.1. Transmission Electron Microscopy

Bright-field transmission electron microscopy (TEM) images were taken at the nanoimaging facility of the chemistry department of the University of Toronto using a Hitachi H-7000 instrument (Hitachi High-Tech Corporation, Tokyo, Japan). Samples were prepared by placing one drop of solution on a Formvar carbon-coated grid, touching the edge of the droplet with a filter paper to remove excess liquid and allowing the grid to dry.

For each sample, micelle length distributions were determined by tracing more than 200 micelles using the software ImageJ (NIH, Laboratory for Optical and Computational Instrumentation, LOCI, University of Wisconsin, Madison, WI, US). Error bars were calculated using the standard error of the mean, s.e.m., obtained with a 99% confidence interval.

### 2.2. Sample Preparation

Six vials, each containing 4 mL of the same seed solution (c = 0.02 mg/mL) were heated at 75 °C in a heating bath. After 40 min of heating, different aliquots (0, 16, 33, 52, 76 and 82 μL) of PFS_53_-*b*-PI_637_ unimer heated in decane (c = 4.8 mg/mL) at 100 °C were added to these solutions. Those solutions were then further annealed at 75 °C for 100, 420, 1200 and 2640 min. After each annealing time, 1 mL of each solution was transferred into empty vials that were also pre-heated to 75 °C. Half (0.5 mL) of each of these solutions were then injected in an empty vial and let to cool to room temperature (23 °C), following the usual self-seeding procedure, while to the second half we added a 5 times excess of PFS_60_-*b*-PDMS_660_ unimers that was pre-heated in decane at 100 °C. This second set of samples was briefly swirled and let at 75 °C for 5 more minutes to fully mix PFS_60_-*b*-PDMS_660_ with the PFS_53_-*b*-PI_637_ unimers remaining in solution. The samples were then removed from the heating bath to cool to room temperature. This procedure allowed us to trap the PFS_53_-*b*-PI_637_ surviving seeds with the large excess of PFS_60_-*b*-PDMS_660_ unimer. After two days of aging at room temperature, we added 0.5 mL of decane to each trapped seed solution, followed by 0.1 mL of Karstedt’s catalyst. The samples were let to age one more day and then studied by TEM. In parallel, the control samples were aged at room temperature for two days prior to be imaged by TEM. This led to a total of 48 samples that were imaged by TEM.

Note that to follow the micelle growth kinetics as a function of unimer concentration, we used a PFS_53_-*b*-PI_637_ seed solution in decane prepared for a previous study [36] and that was carefully stored in a sealed container and aged for one year at 23 °C. Over this time, the size (number average length, *L_seed,RT_*, equal to 43.5 nm) and concentration of seed crystallites remained constant (Figure S1), but aging decreased the number of crystallites that dissolved at a given annealing temperature (Figure S2). We attribute the enhanced robustness of the one-year-old seeds to an increase in the crystallinity of the 1D PFS core. To confirm that this long aging time did not affect the mechanism of the seed dissolution, we plotted the length distribution of one-year aged seeds annealed and trapped at 75 °C, and compared it with the length distribution of the freshly prepared seeds annealed and trapped in similar conditions (Figure S3).

### 2.3. Definition of Key Parameters

The parameter *p* represents the ratio of the amount of PFS_53_-*b*-PI_637_ unimer added to the solution at 75 °C (*m_uni,added_*) to the mass of seed crystallites present in the seed solution at 23 °C (*m_seeds,RT_*) prior to heating the solution to 75 °C. *L_ts_*(*p*,*t*) is the number average length of the trapped seeds annealed at 75 °C for different annealing times, while the number average length of the untrapped micelles cooled to 23 °C is defined as *L_mic_*(*p*,*t*).

Seeded growth can be well described by a simple equation [19]:

(1) $$L_{mic}\;=\;\left(\frac{m_{uni}}{m_{seed}}\;+1\right)\;L_{seed}$$

where *L_mic_* and *L_seed_* are the number average lengths of the micelles and the seeds, respectively, while *m_uni_* is the mass of unimer added to the solution and *m_seed_* is the mass of seeds. Despite its apparent simplicity, one can extract a large amount of information from Equation (1), helping us understand some key phenomena related to seeded growth and self-seeding.

## 3. Results and Discussion

**Figure 1:**
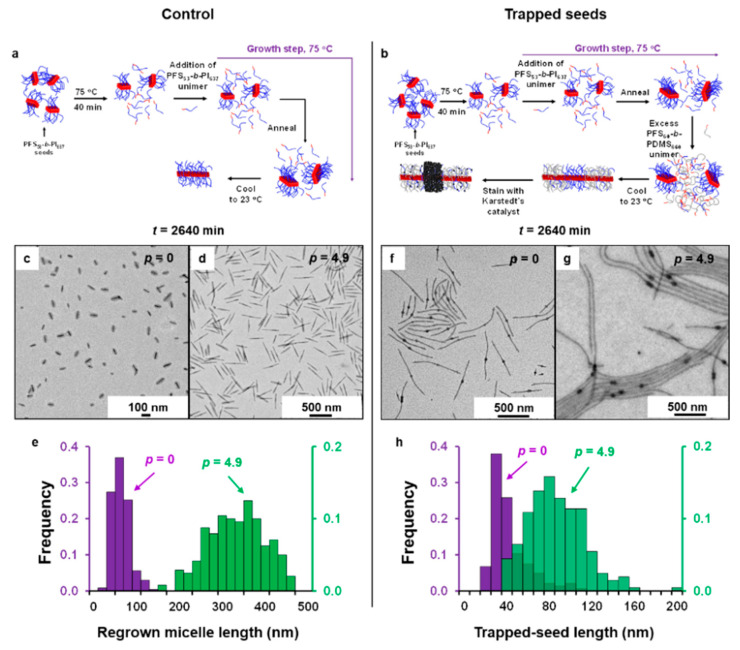
Seed growth kinetics studied by seed trapping. Schematic diagram describing (**a**) the control and (**b**) the seed-trapping experiments performed to study the growth kinetics of PFS_53_-*b*-PI_637_ crystallites annealed at 75 °C for different times in the presence of added PFS_53_-*b*-PI_637_ unimer. In this scheme we use a color code to represent different chemical species: Red represents polyferrocenyldimethylsilane (PFS) (either as the PFS block of a unimer or the crystalline core of a micelle), blue represents polyisoprene (PI), grey represents polydimethylsiloxane (PDMS), while the black spheres represent the platinum nanoparticles from the Karstedt’s catalyst used to stain PI. (**c**,**d**) TEM images of PFS_53_-*b*-PI_637_ micelles obtained by heating seeds at 75 °C for 2640 min, and letting the solution cool to 23 °C, (**c**) without PFS_53_-*b*-PI_637_ unimer added (*p* = 0), and (**d**) in the presence of an initial mass ratio (*p* = 4.9) of PFS_53_-*b*-PI_637_ unimer added to PFS_53_-*b*-PI_637_ seeds. (**e**) Respective histograms of the length distributions of the regrown micelles without (*p* = 0, purple columns), and with unimer added (*p* = 4.9, green columns). (**f**,**g**) TEM images of PFS_53_-*b*-PI_637_ seeds trapped after 2640 min of annealing at 75 °C, (**f**) without PFS_53_-*b*-PI_637_ unimer added (*p* = 0), and (**g**) in the presence of an initial mass ratio of PFS_53_-*b*-PI_637_ unimer added to PFS_53_-*b*-PI_637_ seeds, *p* = 4.9. (**h**) Respective histograms of the length distribution of the surviving PFS_53_-*b*-PI_637_ seeds without (*p* = 0, purple columns), and with unimer added (*p* = 4.9, green columns). Samples (**f**,**g**) were stained with Karstedt’s catalyst to highlight the PI rich regions.

The surviving seeds could easily be delineated by our seed-trapping protocol (Figure 1) [36]. Figure 1f,g shows representative TEM images of the stained trapped seeds after the solution was annealed at 75 °C, for 2640 min without unimer added (*p* = 0, Figure 1f) and with the largest amount of PFS_53_-*b*-PI_637_ unimer added (*p* = 4.9, Figure 1g).

It is important to note that, as shown in a previous study [36], the trapped seeds are ca. 3.5 nm longer than the stained seeds as observed by TEM due to the shrinkage of the corona block induced by the cross-linking of the corona by the karsted’s catalyst.

The effect of the addition of PFS_53_-*b*-PI_637_ unimer on PFS_53_-*b*-PI_637_ seeds can be seen in Figure 1f–h, in Figures S4–S15 and Tables S1 and S2. *L_ts_*(0,2640) is similar to that of the un-aged seeds trapped at room temperature (Figure S16), suggesting that the dissolution of some of the seeds did not induce an obvious growth of the surviving seeds at 75 °C. In the presence of extra unimer (*p* = 4.9, Figure 1g), and after the same annealing time (2640 min), the length distribution of the seeds shifted to larger values, broadened and became Gaussian-like (Figure 1h). However, one sees that even after 2 days of annealing, the length of the trapped seeds remained much smaller than those of the corresponding control samples that were fully regrown at 23 °C (Figure 1c–e), a clear indication that most of the unimer remained in solution at 75 °C.

**Figure 2:**
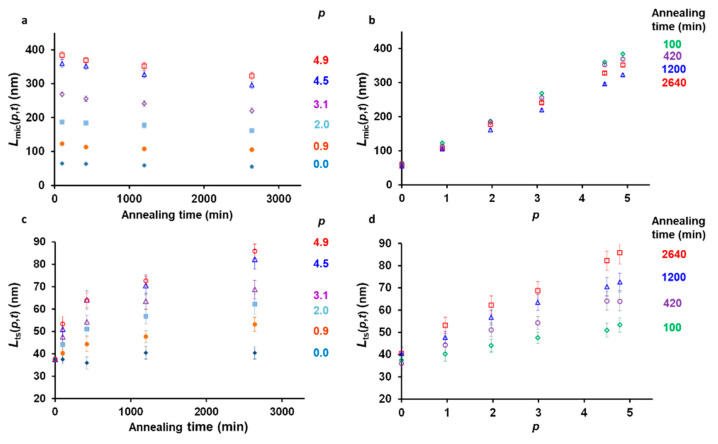
Evolution of the number average lengths of micelle annealed at 75 °C and regrown at room temperature solutions as a function of (**a**) time and (**b**) ratios *p*. Evolution of the number of average lengths of trapped seeds as a function of (**c**) time and (**d**) ratios *p*. *p* is the ratio of the mass of unimer added to the hot seed solution, *m_uni,added_*, to the mass of seeds originally present in the solution, m*_seeds,RT_*. Error bars correspond to the s.e.m. of the length distributions.

Figure 2a shows the evolution of micelles that regrew after annealing (control experiments), *L_mic_*(*p*,*t*), as a function of annealing time for each amount of unimer added to the solution. For all the amounts of unimer added, one sees that the lengths of the micelles regrown at 23 °C decreased with annealing time. This result was rather surprising since one would intuitively expect the lengths of the micelles regrown at 23 °C, *L_mic_*(*p*,*t*) to be independent of annealing time. For each annealing time, however, the lengths of the micelles subsequently regrown at room temperature, *L_mic_*(*p*,*t*) versus *p* obeys Equation (1), still increasedlinearly as a function of the amount of unimer present in solution (Figure 2b).

**Figure 3:**
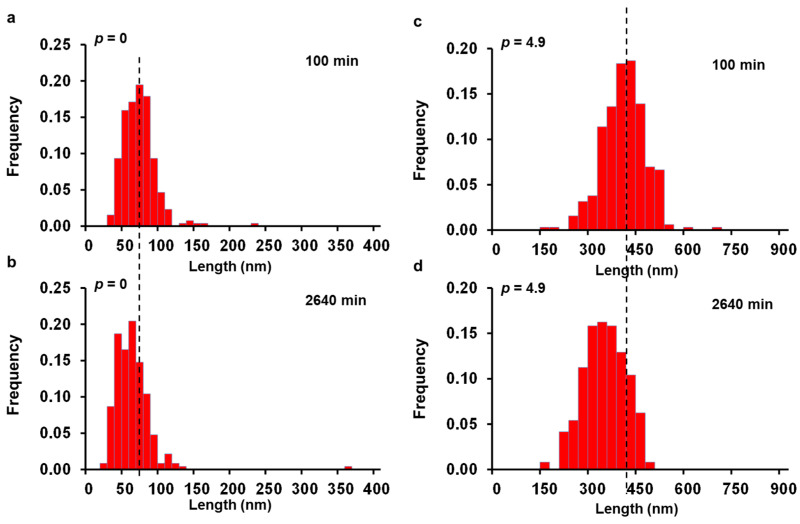
Histograms of the length distributions of PFS_53_-*b*-PI_637_ micelles regrown at room temperature for *p* = 0 after (**a**) 100 min, (**b**) 2640 min, and for *p* = 4.9 after (**c**) 100 min and (**d**) 2640 min of annealing at 75 °C. The vertical dashed lines highlight the shift of the micelle length distributions towards shorter values.

In contrast, the lengths of the seeds trapped at 75 °C, *L_ts_*(*p*,*t*) increased as a function of time (Figure 2c), following much more conventional behavior. Seeded growth was, however, extremely slow since even after two days of annealing, the trapped seeds remained much smaller than the micelles at room temperature, reaching only 90 nm for the largest amount of unimer injected in the solution. The growth kinetics were also non-linear, slowing down with time (Figure S17). Micelle growth, although extremely slow, was still noticeable since, for *p* = 4.9, the trapped seeds were ca. twice longer after 2640 min of annealing than after 100 min. Finally, in Figure 3d we show the plot of the length of the trapped seeds as a function of the different amount of unimer added to the solution for each annealing time. In this plot, one observes that *L_ts_*(*p*,*t*) increased linearly as a function of *p*, as already seen for *L_mic_*(*p*,*t*) versus *p*.

The seed-trapping and control experiments were rather simple to perform, leaving little doubts about the validity of the results obtained. Interestingly, their combination coupled with the use of Equation (1) (which is also highly straightforward) can unravel complex phenomena, as shown in the following sections.

### 3.1. Control Experiments: Lateral Growth versus Fragmentation during Annealing

The number average lengths of the seeds annealed at 75 °C for different times (100, 420, 1200 and 2640 min) (Figure S18) and cooled to room temperature decreased monotonically from ca. 64.4 nm for the sample annealed for 100 min down to 55.4 nm when annealed for 2640 min. Although the length distributions of the sample shifted toward lower values (Figure 3a,b), the variation was quite small and could be considered, a priori, as part of the experimental error. However, similar behavior was observed for all the control samples, becoming increasingly noticeable as the amount of unimer added to the solution increased (Figure 3c,d), pointing toward a systematic effect of the annealing time on the final length of the micelles regrown at room temperature.

Previous works have shown that two main phenomena could explain this behavior:

(a) Unimer chains could add laterally onto the micelles during annealing. These chains would thus not participate to the elongation of the micelles. Since lateral growth is expected to be time dependent, the amount of unimer in solution that could participate to the elongation of the micelle at 23 °C would decrease with time.

(b) The micelles could fragment with time, increasing the number of seeds in solution which would lead to a decrease in the lengths of the micelles once they regrew at 23 °C.

The one-year aged seed crystallites were much more stable towards annealing at 75 °C than their freshly prepared counterpart (Figure S2). Therefore, one can conclude that this long aging time favored the packing of the crystalline core and decreased the distance between two grafting points. As a result, the densification of the micelle crystalline core would lead to an increase in their grafting density, hindering the lateral growth of the micelles [38]. We thus assume that the decrease in *L_mic_*(*p*,*t*) as a function of annealing time is mainly due to seed fragmentation, while lateral growth can be neglected. We verified these assumptions by evaluating the ratio *L_mic_*(*p*,*t*)/*L_mic_*(0,*t*) as a function of annealing time for both scenarios, and comparing it with the experimental plot of *L_mic_*(*p*,*t*)/*L_mic_*(100,*t*) versus time shown in Figure 2b.

The lateral growth of the micelles during annealing would lead to an increase in the linear aggregation number of the micelles in the section that was regrown. Therefore, we can rewrite Equation (1) to express *L_mic_*(*p*,*t*) as a function of both *p* and *t*:

(2) $$L_{mic}\left(p,t\right)\;=\;\left(\frac{N_{agg/L}\left(p,t\right)}{N_{agg/L,RT}}\frac{m_{uni}\left(p,t\right)}{m_{ts}\left(p,t\right)}\;+\;1\right)\;L_{ts}\left(p,t\right)\;$$

where *N_agg/L_*(*p*,*t*), is the linear aggregation number of the trapped seeds, *N_agg/L,RT_* is the linear aggregation number of the seeds at room temperature. *m_uni_*(*p*,*t*) is the amount of unimer that is still present in solution after it was annealed at 75 °C for a time *t*.

We recall that *m_seeds,RT_* = *m_uni_*(0,0) + *m_ts_*(0,0), while by definition, *m_added_*(*p*,0) = *p m_seeds,RT_*, where *m_uni_*(0,0) is the mass of unimer coming from the dissolution of some of the starting seeds, *m_added_*(*p*,0) is the mass of unimer added to the solution and *m_ts_*(0,0) is the mass of the surviving seeds just after heating. Equation (2) thus gives (see Supporting discussion, Section I):

(3) $$L_{mic}\left(p,t\right)\;=L_{ts}\left(0,0\right)\left[\left(p+1\right)\;\frac{m_{seeds,RT}}{m_{ts}\left(0,0\right)}\right]\;+\;L_{ts}\left(p,t\right)\left[1-\;\frac{N_{agg/L}\left(p,t\right)}{N_{agg/L,RT}}\right]$$

For *t* = 0, *N_agg/L_*(*p*,0) = *N_agg/L,RT_* (the linear aggregation number does not change), leading to:

(4) $$L_{mic}\left(p,0\right)\;=\;\frac{m_{seed,RT}}{m_{ts}\left(0,0\right)}\left(p+1\right)\;L_{ts}\left(0,0\right)$$

Here, we note that for the specific case where *p* and *t* are equal to 0, Equation (4) gives:

(5) $$\;\frac{m_{seeds,RT}}{m_{ts}\left(0,0\right)}\;=\frac{L_{mic}\left(0,0\right)}{L_{ts}\left(0,0\right)}$$

Therefore, Equation (3) becomes:

(6) $$\frac{L_{mic}\left(p,t\right)}{L_{mic}\left(p,0\right)}\;=1-\;\frac{\;L_{ts}\left(p,t\right)}{\;L_{mic}\left(0,0\right)\;}\left(\frac{N_{agg}\left(p,t\right)-N_{agg,RT}}{N_{agg,RT}}\right)\frac{1}{\left(p+1\right)}$$

On the other hand, if the decrease of *L_mic_*(*p*,*t*) as a function of time is solely due to the fragmentation of the seeds as a function of time (and, thus to an increase in the number of seeds), we can show (Supporting discussion, Section II) that:

(7) $$L_{mic}\left(p,t\right)\;=\;\frac{m_{seed,RT}}{m_{ts}\left(0,0\right)}\left(p+1\right)\;L_{ts,f}\left(p,t\right)$$

where *L*_ts,f_(*p*,*t*) is the length of the fragmented seeds as a function of annealing time. 

We note that Equation (7) is equivalent to Equation (4), using *L_ts,f_*(*p*,*t*) instead of *L_ts_*(*p*,*t*). At time, *t* = 0, just after the seed solution reached 75 °C, although the seeds may have fragmented, they did not grow, thus, the length of the fragmented seeds at *t* = 0 is the same as the length of the trapped seeds, i.e., *L_ts,f_*(*p*,0) = *L_ts_*(*p*,0) = *L_ts_*(0,0). We can thus write:

(8) $$\frac{L_{mic}\left(p,t\right)}{L_{mic}\left(p,0\right)}\;=\;\;\frac{L_{ts,f}\left(p,t\right)}{L_{ts}\left(0,0\right)}$$

Equations (6) and (8) indicate that the evolution of *L_mic_*(*p*,*t*)/*L_mic_*(100,*t*) versus time for different values of *p* strongly depends on the annealing history. If lateral growth occurs during annealing (Equation (6)) then, one should expect *L_mic_*(*p*,*t*)/*L_mic_*(100,*t*) to vary with the amount of unimer added to the solution, while in the case of seed fragmentation (Equation (8)), *L_mic_*(*p*,*t*)/*L_mic_*(100,*t*) would be independent of *p* if the seed fragmentation does not depend on the amount of unimer added to the solution.

**Figure 4:**
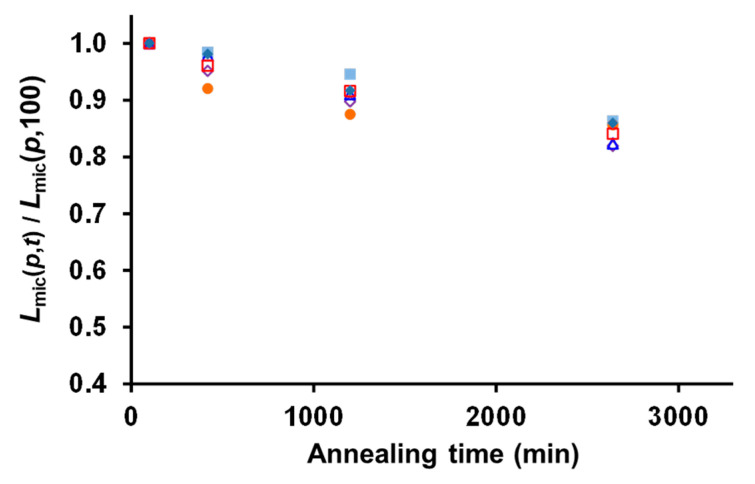
Plot of [*L_mic_*(*p*,*t*)/*L_mic_*(*p*,100)] as a function of annealing time for the different ratios of the mass of unimer added to the hot seed solution, *m_uni,added_*, to the mass of seeds originally present in the solution, m*_seeds,RT_* (*p* = 0, 0.9, 2, 3.1, 4.5, 4.9).

To evaluate which equation better describe the experimental results, we plotted *L_mic_*(*p*,*t*)/*L_mic_*(100,*t*) versus time for the different amount of unimer added to the solution (Figure 4). In this plot, *L_mic_*(*p*,*t*)/*L_mic_*(100,*t*) appears to be independent on the amount of unimer injected into the seed crystallite solution for the annealing times investigated, a strong indication that the change in the final lengths of the micelles for the control experiment is mainly due to seed fragmentation (additional discussion can be found in Supporting Information).

### 3.2. Fragmentation versus Annealing Time

The presence of fragmentation could strongly affect the micelle growth kinetics. Indeed, the impact of fragmentation on the micelle lengths during seeded growth is multifold. It decreases the length of the micelles that fragment, and it increases the number of seeds the unimer can add on. Fragmentation could also influence the growth kinetics. For example, if seeded growth was a diffusion limited process, the increase in the number of seeds in solution would decrease the diffusion time between the unimer and the seed ends, which would lead to an increase in growth kinetics. It might thus appear difficult to quantify the effect of fragmentation on micelle growth.

Fortunately, we can use some key observations that were made in previous reports as well as in the present work to simplify the equations. Boott et al. [11] have shown that the seeded growth of PFS_63_-*b*-PDMS_513_ (a system similar to that presented in this study) was not diffusion-limited. We can thus assume that the growth kinetics is not influenced by the seed fragmentation. In addition, the overlapping plots of *L_mic_*(*p*,*t*)/*L_mic_(p*,100) indicate that micelle fragmentation does not depend on the amount of unimer present in solution. Finally, we distinguish dissolution from fragmentation, in the sense that fragmentation leads to the formation of more micelles/seeds, but does not add any unimer in solution.

The number average length of a population of *N* seeds is given by:

(9) $$L_{n}\;=\;\;\frac{\sum_{i=1}^{N}L_{i}}{N}$$

where *L_i_* is the length of seed *i*, and *N* is the total number of seeds. If *z* new seeds are formed via fragmentation, the number average length of the seed solution, *L_n,f_* becomes:

(10) $$L_{ts,f}\left(t\right)\;=\;\;\frac{\sum_{i=1}^{N+f}L_{i}^{’}\left(t\right)}{N+z\left(t\right)}=\frac{\sum_{i=1}^{N}L_{i}}{N+z\left(t\right)}=\frac{\sum_{i=1}^{N}L_{i}}{N}\frac{1}{1+z\left(t\right)/N}=L_{ts}\left(0,0\right)\;f\left(t\right)$$

where *L*′_i_ is the length of seed *i*, after fragmentation. It is important to note that Equation (10) is only correct in absence of dissolution during the fragmentation, since in this case, the total length of the seeds is unchanged and *z*(*t*)/*N* increases with time, from 0 to a finite positive value.

In the present work, seed annealing can be schematized as a two-step process. First, as soon as the annealing temperature was reached, the seeds with the lowest crystallinity dissolved [36], while the rest of the seeds survived. The dissolution step can be considered instantaneous. In the second step, the seeds fragmented. As shown in Figure 2a, Figure 3 and Figure 4, this step is time dependent.

The difficulty here resides in choosing a physically meaningful equation that would describe the fragmentation of the trapped seeds during annealing. In a previous work [36], we showed that short seeds, such as those used in this study, would mainly fragment in the center until they reach a critical length, *L_c,f_* [40,41,42,43], that was estimated to be close to 32 nm. Since we are using the same seed solution, we would expect the critical length to also be close to 32 nm.

It is also important to note that the increase in length of the seeds during annealing at 75 °C (Figure 2c,d), did not affect their fragmentation, since the plots of *L_mic_*(*p*,*t*)/*L_mic_*(100,*t*) versus time (Figure 4) overlap for all the values of *p*. This result, in apparent contradiction with previous observations [44], suggests that the original seeds were more fragile than their extended counterparts. This phenomenon may find its origin in the fact that the original seeds were grown at room temperature, while the extended parts were grown at 75 °C, which could facilitate a better packing of the crystalline block, strengthening the micelle core.

From this description, we infer that the evolution of the number of fragmentation events as a function of time could be approximated by a normal distribution centered at *t* = 0. Indeed, at short time, the number of seeds that would fragment might be relatively large, but as the annealing time increases, the seeds would strengthen, and the number of fragmentation events would slowly decrease. Since the number of seeds increases at each fragmentation event, one needs to consider the sum of all the events as a function of time, leading to a cumulative distribution function:

(11) $$z\left(t\right)\;=\;2\;\overset{t}{\underset{0}{\int }}\frac{\exp\left(-\frac{x^{2}}{2\;\sigma^{2}}\right)}{\sqrt{2\pi \sigma^{2}}}dx\left[\alpha -1\right]\;N$$

and

(12) $$f\left(t\right)=\frac{1}{1+z\left(t\right)/N}=\frac{1}{1+2\;\overset{t}{\underset{0}{\int }}\frac{\exp\left(-\frac{x^{2}}{2\;\sigma^{2}}\right)}{\sqrt{2\pi \sigma^{2}}}dx\left[\alpha -1\right]\;}$$

where *σ* can be related to the rate of fragmentation, since fast fragmentation would lead to a low value of *σ*, while *α* is a normalization factor defined as:

(13) $$\alpha =\;\frac{L_{ts}(0,0)}{L_{c,f}}$$

If the length of the surviving seeds at *t* = 0 is equal to the critical length, *L_c,f_*, then *α* = 1, and the seeds will not fragment. However, if *α* is large, then the decrease in seed lengths due to fragmentation will also be large.

From Equation (10), the change of the trapped seed lengths as a function of fragmentation is thus:

(14) $$L_{ts,f}\left(0,t\right)\;=\;\;\frac{L_{ts}(0,0)}{2\;\overset{t}{\underset{0}{\int }}\frac{\exp\left(-\frac{x^{2}}{2\;\sigma^{2}}\right)}{\sqrt{2\pi \sigma^{2}}}dx\left[\alpha -1\right]+1}$$

Since the amount of unimer added to the seed solution did not affect their fragmentation, we can write the more general equation:

(15) $$L_{ts,f}\left(p,t\right)\;=\;\;\frac{L_{ts}(0,0)}{2\;\overset{t}{\underset{0}{\int }}\frac{\exp\left(-\frac{x^{2}}{2\;\sigma^{2}}\right)}{\sqrt{2\pi \sigma^{2}}}dx\left[\alpha -1\right]+1}$$

with *L_ts_*(0,0) = *L_ts_*(*p*,0), since at time *t* = 0, no unimer would have time to add onto the surviving seeds.

Incorporating Equation (15) into Equation (7) leads to:

(16) $$L_{mic}\left(p,t\right)\;=\;\left(p+1\right)\;\frac{L_{mic}\left(0,0\right)}{2\;\overset{t}{\underset{0}{\int }}\frac{\exp\left(-\frac{x^{2}}{2\;\sigma^{2}}\right)}{\sqrt{2\pi \sigma^{2}}}dx\left(\frac{L_{ts}(0,0)}{L_{c,f}}-1\right)+1}$$

**Figure 5:**
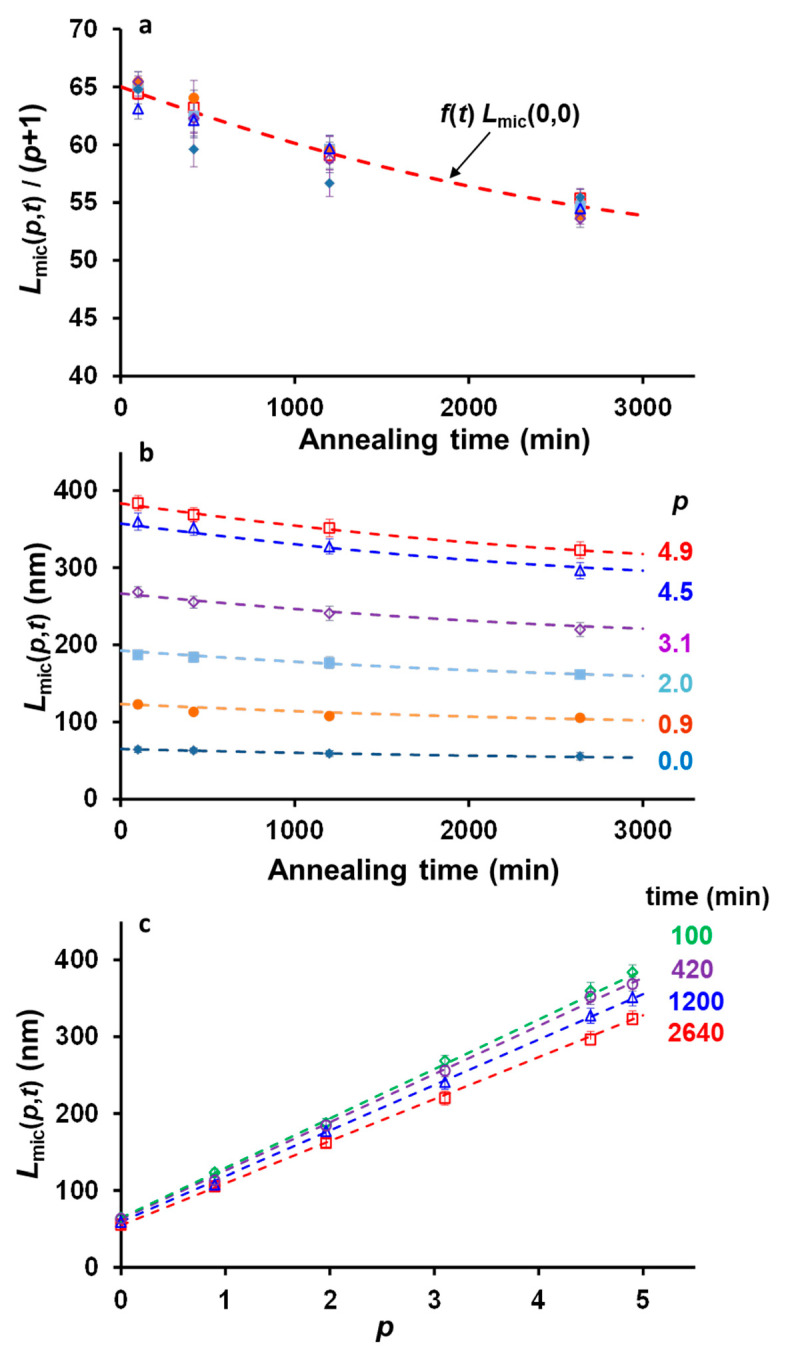
(**a**) Plot of [*L_mic_*(*p*,*t*)/(1 + *p*)] as a function of annealing time (symbols). Fitting Equation (16) to these data (dashed line) led to the values of *σ* = 2700 min, *L_mic_*(0,0) = 65 nm, *L_ts_*(0,0) = 41 nm and *L_c,f_* = 32 nm. Plots of the length of the regrown micelles, *L_mic_*(*p*,*t*), fitted to Equation (16) (dashed lines) as a function of (**b**) time and (**c**) *p*. Error bars correspond to the s.e.m. of the length distributions.

Equation (16) can thus be used to fit *L_mic_*(*p*,*t*)/(1 + *p*) versus time (Figure 5a). For this fit, we used the values of *σ* = 2700 min, *L_mic_*(0,0) = 65 nm, *L_ts_*(0,0) = 41 nm and *L_c,f_* = 32 nm, which is the value expected from the superblob approach [36]. In a previous work, we have shown that in dilute solution seeds dissolve in a cooperative (explosive) process. Thus, seeds that survive dissolution would be expected to have a length similar to the original seeds, i.e., 43.5 nm, close to the value of *L_ts_*(0,0) = 41 nm used to fit the data.

Fits of *L_mic_*(*p*,*t*) as a function of *p* and *t* are shown in Figure 5b,c. Despite the inherent uncertainty in the measurements of the micelle lengths, we could fit reasonably well the experimental data shown in Figure 5b,c using Equation (16).

### 3.3. Growth Kinetics at 75 °C

Equation (16) gives the evolution of the number average lengths of the micelles once they were fully regrown at room temperature. The lengths of these micelles were only dependent on the number of seeds present in solution, i.e., seed fragmentation. Growth kinetics could thus be ignored.

The situation is quite different for the seeds trapped at 75 °C, since the lengths of the micelles as a function of time depend on both micelle fragmentation and growth kinetics. Figure 2c,d, shows that the trapped seeds annealed for 2640 min were twice longer when *p* = 4.9 than in absence of unimer added to the solution (*p* = 0). The master curve obtained for *L_mic_*(*p*,*t*)/(*p* + 1) versus time (Figure 5a), however, suggests that fragmentation did not depend on the amount of unimer added. From these two observations, we conclude that fragmentation and micelles growth were independent from each other, in our experimental conditions. Indeed, if fragmentation was related to micelle growth, then a larger amount of micelles would have fragmented when the micelles were longer, and *L_mic_*(*p*,*t*)/(*p* + 1) would have varied with *p*. Therefore, fragmentation and growth can be seen as two independent time functions. We can thus write:

(17) $$L_{ts}\left(p,t\right)=\;f(t)\;g\left(p,t\right)$$

where *f*(*t*) is the fragmentation function given in Equation (12), while *g*(*p*,*t*) describes micelle growth as a function of *p* in absence of fragmentation:

(18) $$g\left(p,t\right)=\left[\frac{m_{uni}\left(p,0\right)-m_{uni}\left(p,t\right)\;}{m_{ts}\left(p,0\right)}+1\right]L_{ts}\left(p,0\right)$$

with *L_ts_*(*p*,0) = *L_ts_*(0,0), and *m_ts_*(*p*,0) = *m_ts_*(0,0).

As shown in Supporting Information, taking into account the effect of the molecular weight distribution of PFS_53_-*b*-PI_637_, leads to:

(19) $$g(p,t)=\;\;L_{ts}(0,0)\left[1+\;\left(\frac{L_{mic}\left(0,0\right)}{L_{ts}\left(0,0\right)}\left(1+p\right)-1\right)\left(1-\;e^{-{\left(k^{*}t\right)}^{\beta }}\right)\right]$$

where k* is the growth rate constant and *β*, the stretching exponent.

A stretched exponential is a signature of a distribution of growth rates [45,46]. This kind of distribution could arise if the deposition rate of a unimer were highly sensitive either to the length of its PFS block or the block ratio of the BCP. Even though PFS_53_-*b*-PI_637_ has a narrow molecular weight distribution (Ð = 1.01), it is not monodispersed. The stretched exponential fit suggests that fractionation affects the micelle growth at 75 °C [47]. This result is consistent with the observation made in one of the rare studies of the fractionation of narrowly dispersed BCP by crystallization [48]. It is also in agreement with the recent work from Song et al., who studied the CDSA of crystalline-coil BCPs with corona-forming block of various molecular weight distributions [49].

Incorporating Equations (16) and (19) into Equation (17) finally gives the growth kinetics of the micelles at 75 °C in the presence of fragmentation.

(20) $$L_{ts}\left(p,t\right)=\;\frac{L_{ts}(0,0)\left[1+\;\left(\frac{L_{mic}\left(0,0\right)}{L_{ts}\left(0,0\right)}\left(1+p\right)-1\right)\left(1-\;e^{-{\left(k^{*}t\right)}^{\beta }}\right)\right]}{2\;\overset{t}{\underset{0}{\int }}\frac{\exp\left(-\frac{x^{2}}{2\;\sigma^{2}}\right)}{\sqrt{2\pi \sigma^{2}}}dx\left(\frac{L_{ts}(0,0)}{L_{c,f}}-1\right)+1}$$

**Figure 6:**
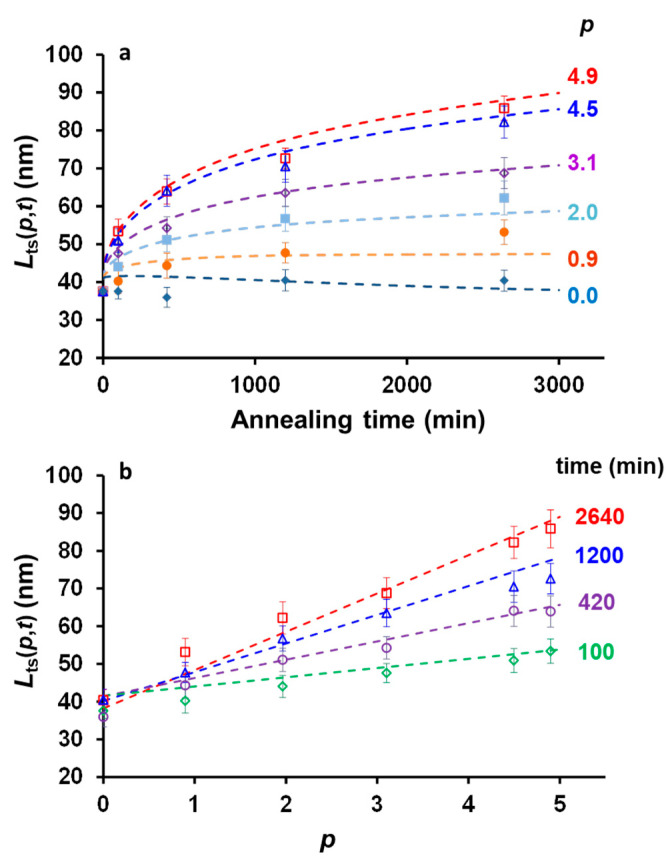
Plot of the of the length of the trapped crystallites, *L_ts_*(*p*,*t*) fitted to Equation (20) as a function of (**a**) time, and (**b**) *p*.

Fits of *L_ts_*(*p*,*t*) as a function of *p* and *t* are shown in Figure 6. To fit the growth kinetics data (Figure 6a,b), we used k* = 1.7 × 10^−5^ min^−1^ and *β* = 0.51. Interestingly, these values compared well with the data obtained by Boott et al. [11] for the growth of PFS_63_-*b*-PDMS_513_ in *n*-hexane at different temperatures. Indeed, extrapolation of the Eyring plot (ln(*k*′/T) versus 1/T) that they generated from kinetic data obtained at different temperatures led to a rate constant *k*′ = 4.4 × 10^−5^ min^−1^ (or *k*′ = 7.3 × 10 ^−7^ s^−1^), which is in the same order of magnitude as the rate constant deduced from Equation (20).

### 3.4. Evaluation of the Growth Rate as a Function of Unimer Concentration

Equation (20) gives us the possibility to evaluate the growth rate of the core crystalline micelles as a function of unimer concentration:

(21) $$G\left(p,t\right)=\frac{\partial \;L_{ts}(p,t)}{\partial \;t}$$

By itself, *G*(*p*,*t*) is extremely complex. We are, however, interested in evaluating the variation of *G*(*p*,*t*) as a function of *p*. For this reason, one simply needs to rewrite Equation (20) to isolate all the terms that depend on *p*:

(22) $$L_{ts}\left(p,t\right)=\;\frac{L_{ts}\left(0,0\right)e^{-{\left(k^{*}t\right)}^{\beta }}+L_{mic}\left(0,0\right)\left(1-\;e^{-{\left(k^{*}t\right)}^{\beta }}\right)}{f\left(t\right)}+p\frac{L_{mic}\left(0,0\right)\left(1-\;e^{-{\left(k^{*}t\right)}^{\beta }}\right)}{f\left(t\right)}=x\left(t\right)+p\;y\left(t\right)$$

Therefore *G*(*p*,*t*) is given by:

(23) $$G\left(p,t\right)=\;\frac{d\;x\left(t\right)}{d\;t}+p\;\frac{d\;y\left(t\right)}{d\;t}$$

*G*(*p*,*t*) thus increases linearly with *p*, and since *p* is proportional to the unimer concentration, *c*, we can write that *G* ∝ *c^γ^*, with *γ* = 1. This special case indicates that the micelle growth proceeded in the mononucleation regime, where one nucleus adds at a time on the growth face. The linear increase of *G*(*p*,*t*) with *p* is in agreement with the results of Monte Carlo simulations reported by Hu et al. [50,51] for planar growth of 1D crystals.

## 4. Conclusions

In summary, we have demonstrated that a seed-trapping protocol can be used to investigate the crystal growth kinetics of 1D micelles at elevated temperatures where both seed dissolution and fragmentation happened. Seed trapping proved particularly efficient in delineating the surviving seeds, allowing us to measure them after different annealing time. By comparing the length of the trapped seeds with the lengths of micelles that followed the exact same thermal history without being trapped, we could develop a kinetic model that accounted for seed fragmentation during crystallite growth. We considered that the probability that a seed fragment was decreasing with time, following a normal distribution function. The increase in number of seeds in solution could thus be described by a cumulative distribution function that is not dependent on the amount of unimer present in solution, and thus on the seeded growth kinetics. As previously reported, we observed that the seeded growth kinetics could be well modeled by a stretched exponential, which we believe is due to the fractionation by crystallization of the narrowly dispersed BCP [52]. Finally, we used our growth kinetics model to evaluate the variation of the growth rate as a function of the amount of unimer present in solution. We found that the 1D growth of the crystallites occurs in the mononucleation regime.

This study shows how seed trapping can be applied to study 1D micelle growth of other crystalline-coil BCPs, as well as more complex systems such as the parallel growth of two or three 1D crystals from a single BCP crystal face [53].

## Supplementary Materials

The following are available online at https://www.mdpi.com/article/10.3390/polym13183122/s1. Supporting discussion Section I: Effect of lateral growth on *L*_mic_(*p*,*t*) during annealing. Supporting discussion Section II: Effect of fragmentation on *L*_mic_(*p*,*t*) during annealing. Supporting discussion Section III: Growth kinetics at 75 °C. Table S1: Values of *L*_mic_(*p*,*t*) of the PFS_53_-*b*-PI_637_ crystallites heated for different annealing times at 75 °C and cooled to room temperature (control experiments), Table S2: Values of *L*_ts_(*p*,*t*) of the PFS_53_-*b*-PI_637_ crystallites heated for different annealing time at 75 °C (seed-trapping experiments), Figure S1: Effect of PFS53-b-PI637 seed crystallite history on their stability at RT, Figure S2: Effect of PFS53-b-PI637 seed crystallite history on their stability against dissolution upon heating at 75 °C, Figure S3: Comparison of the histograms of the length distribution PFS53-b-PI637 seed crystallites that were trapped with PFS_60_-*b*-PDMS_660_ unimer after being heated at 75 °C, Figure S4: TEM micrographs of PFS_53_-*b*-PI_637_ seeds trapped after ((a) 100 min, ((b) 420 min, ((c) 1200 min and (d) 2640 min of annealing at 75 °C without PFS_53_-*b*-PI_637_ unimer added (*p* = 0), Figure S5: TEM micrographs of PFS_53_-*b*-PI_637_ seeds trapped after (a) 100 min, (b) 420 min, (c) 1200 min and (d) 2640 min of annealing at 75 °C in the presence of an initial mass ratio of PFS_53_-*b*-PI_637_ unimer added to PFS_53_-*b*-PI_637_ seeds *p* = 0.9, Figure S6: TEM micrographs of PFS_53_-*b*-PI_637_ seeds trapped after ((a) 100 min, ((b) 420 min, ((c) 1200 min and ((d) 2640 min of annealing at 75 °C in the presence of an initial mass ratio of PFS_53_-*b*-PI_637_ unimer added to PFS_53_-*b*-PI_637_ seeds *p* = 2, Figure S7: TEM micrographs of PFS_53_-*b*-PI_637_ seeds trapped after ((a) 100 min, ((b) 420 min, ((c) 1200 min and (d() 2640 min of annealing at 75 °C in the presence of an initial mass ratio of PFS_53_-*b*-PI_637_ unimer added to PFS_53_-*b*-PI_637_ seeds *p* = 3.1, Figure S8: TEM micrographs of PFS_53_-*b*-PI_637_ seeds trapped after ((a) 100 min, ((b) 420 min, (c) 1200 min and (d) 2640 min of annealing at 75 °C in the presence of an initial mass ratio of PFS_53_-*b*-PI_637_ unimer added to PFS_53_-*b*-PI_637_ seeds *p* = 4.5, Figure S9: TEM micrographs of PFS_53_-*b*-PI_637_ seeds trapped after (a) 100 min, (b) 420 min, (c) 1200 min and (d) 2640 min of annealing at 75 °C in the presence of an initial mass ratio of PFS_53_-*b*-PI_637_ unimer added to PFS_53_-*b*-PI_637_ seeds *p* = 4.9, Figure S10: Histograms of the length distributions of PFS_53_-*b*-PI_637_ trapped seeds after (a) 100 min, (b) 420 min, (c) 1200 min and (d) 2640 min of annealing at 75 °C without PFS_53_-*b*-PI_637_ unimer added (*p* = 0), Figure S11: Histograms of the length distributions of PFS_53_-*b*-PI_637_ trapped seeds after (a) 100 min, (b) 420 min, (c) 1200 min and (d) 2640 min of annealing at 75 °C in the presence of an initial mass ratio of PFS_53_-*b*-PI_637_ unimer added to PFS_53_-*b*-PI_637_ seeds *p* = 1, Figure S12: Histograms of the length distributions of PFS_53_-*b*-PI_637_ trapped seeds after (a) 100 min, (b) 420 min, (c) 1200 min and (d) 2640 min of annealing at 75 °C in the presence of an initial mass ratio of PFS_53_-*b*-PI_637_ unimer added to PFS_53_-*b*-PI_637_ seeds *p* = 2, Figure S13: Histograms of the length distributions of PFS_53_-*b*-PI_637_ trapped seeds after (a) 100 min, (b) 420 min, (c) 1200 min and (d) 2640 min of annealing at 75 °C in the presence of an initial mass ratio of PFS_53_-*b*-PI_637_ unimer added to PFS_53_-*b*-PI_637_ seeds *p* = 3, Figure S14: Histograms of the length distributions of PFS_53_-*b*-PI_637_ trapped seeds after (a) 100 min, (b) 420 min, (c) 1200 min and (d) 2640 min of annealing at 75 °C in the presence of an initial mass ratio of PFS_53_-*b*-PI_637_ unimer added to PFS_53_-*b*-PI_637_ seeds *p* = 4.5, Figure S15: Histograms of the length distributions of PFS_53_-*b*-PI_637_ trapped seeds after (a) 100 min, (b) 420 min, (c) 1200 min and (d) 2640 min of annealing at 75 °C in the presence of an initial mass ratio of PFS_53_-*b*-PI_637_ unimer added to PFS_53_-*b*-PI_637_ seeds *p* = 4.8, Figure S16: Histograms of the length of PFS_53_-*b*-PI_637_ seed crystallites in decane that were trapped with added PFS_60_-*b*-PDMS_660_ unimer after being heated at 75 °C, then cooled to RT and stained with Karstedt’s catalyst, Figure S17. Evolution of the number average lengths of the number average lengths of trapped seeds as a function of time for *p* = 0, 0.9, 2, 3.1, 4.5 and 4.9, Figure S18: Evolution of the number average lengths of PFS_53_-*b*-PI_637_ seed crystallites annealed in decane at 75 °C, and cooled to 23 °C, as a function of the annealing time (control experiment), Figure S19: Plot of *L_mic_*(*p*,*t*) as a function of (1 + *p*) *L_mic_*(100,*t*).
