# Supplementary material for: In-Depth Analysis of the Effect of Fragmentation on the Crystallization-Driven Self-Assembly Growth Kinetics of 1D Micelles Studied by Seed Trapping
Source: Polymers (Basel). 2021 Sep 16;13(18):3122. doi: 10.3390/polym13183122 (PMC8472273; doi:10.3390/polym13183122)
Supplement: Supplementary file 1 [file polymers-13-03122-s001.zip › polymers-1375324-supplementary.pdf]

Supplementary Materials

In-depth analysis of the effect of fragmentation on the crystallization-driven self-assembly growth kinetics of 1D micelles studied by seed trapping.

Gerald Guerin,* Paul A. Rupar and Mitchell A. Winnik*

Section I: Effect of lateral growth on $L_{mic}(p,t)$ during annealing

We have seen in previous works that when seeds were annealed at high temperature, they could undergo lateral growth via addition of unimer onto the sides of the micelle crystalline core. This broadening led to an increase in the micelle linear aggregation number, defined as the number of polymer molecules per unit length. [1]

To understand the impact of the seed broadening on the amount of unimer in solution during annealing, we first recall the equation that relates the mass of the surviving seeds just after they reached the annealing temperature, $m_{ts}(0,0)$, to the linear aggregation number of these seeds at room temperature, $N_{agg/L,RT}$:

$$m_{ts}(0,0) = N_{ts}(0,0) N_{agg/L,RT} M_0 L_{ts}(0,0) \quad \text{(S1)}$$

Where M_0 is the weight average molecular weight of the diblock copolymer, $N_{ts}(0,0)$ is the number of surviving seeds, and $L_{ts}(0,0)$ is the number average length of the surviving seeds when no unimer was added to the solution (p = 0) at time t = 0. Since the extra unimer solution was added after the seed solution reached the annealing temperature, we can also consider that $m_{ts}(p,0)$ = $m_{ts}(0,0)$, $N_{ts}(p,0) = N_{ts}(0,0)$ and $L_{ts}(p,0) = L_{ts}(0,0)$. In other words, the addition of unimer to the seed solution did not affect the number and length of the surviving seeds at the beginning of annealing.

To further evaluate the effect of broadening during annealing on the seed lengths, we need first to write the equation of the seeded growth of micelles with different linear aggregation numbers. Indeed, equation 1, main text, only gives the simplest equation in the case where the linear aggregation number of the extended section of the seeds is the same as that of the starting seeds, which would not be the case when seed broadening happens.

When the linear aggregation number of the extended part of the seeds, $N_{agg,ext}$, is larger than that of the starting seed, $N_{agg,seed}$, we have from equation 1, main text:

$$L_{mic} = \left(\frac{N_{agg,seed}\, m_{uni}}{N_{agg,ext}\, m_{seed}} + 1 \right) L_{seed} \quad \text{(S2)}$$

However, if the unimer adds both on the side and on the ends of the starting seeds then equation S2 becomes:

$$L_{mic} = \left(\frac{N_{agg,seed}}{N_{agg,ext}} \frac{m_{uni}}{m_{seed}} + 1 \right) L_{seed} - L_{seed} + L_{seed} \frac{N_{agg,seed}}{N_{agg,ext}} \tag{S3a}$$

which gives:

$$L_{mic} = \left(\frac{m_{uni}}{m_{seed}} + 1 \right) L_{seed} \frac{N_{agg,seed}}{N_{agg,ext}} \tag{S3b}$$

If the seeds broadened during annealing, we can thus use equation S3b to describe the seeded growth at this annealing temperature:

$$L_{ts}(p,t) = \left(\frac{m_{uni}(p,0) - m_{uni}(p,t)}{m_{ts}(0,0)} + 1 \right) L_{ts}(0,0) \frac{N_{agg/L,RT}}{N_{agg/L}(p,t)} \tag{S4}$$

where $N_{agg/L}(p,t)$ is the linear aggregation number of the seeds during annealing at 75 ^{o}C for a time t, and $m_{uni}(p,t)$ is the mass of unimer that was still present in solution after the seed solution was annealed at 75 ^{o}C for a time t, while $m_{uni}(p,0)$ is the amount of unimer in solution just after the solution reached the annealing temperature.

Once the solution is cooled to room temperature, the unimer that did not grow onto the seeds during annealing added on the annealed seeds. However, the linear aggregation number of this regrown section is the linear aggregation of the seeds at room temperature due to corona crowding effects [2]. The length of the regrown micelles, $L_{mic}(p,t)$ can thus be written as a function of the length of the seeds annealed for a time t:

$$L_{mic}(p,t) = \left(\frac{N_{agg/L}(p,t)}{N_{agg/L,RT}} \frac{m_{uni}(p,t)}{m_{ts}(p,t)} + 1 \right) L_{ts}(p,t) \tag{S5}$$

We can then incorporate equation S4 into equation S5:

$$L_{mic}(p,t) = \left(\frac{N_{agg/L}(p,t)}{N_{agg/L,RT}} \frac{m_{uni}(p,t)}{m_{ts}(p,t)} + 1 \right) \left(\frac{m_{uni}(p,0) - m_{uni}(p,t)}{m_{ts}(0,0)} + 1 \right) L_{ts}(0,0) \frac{N_{agg/L,RT}}{N_{agg/L}(p,t)} \tag{S6}$$

The mass of unimer that grew onto the seeds during annealing is given by:

$$m_{uni}(p,0) - m_{uni}(p,t) = m_{ts}(p,t) - m_{ts}(0,0) \tag{S7}$$

Incorporating S7 into S6 leads to:

$$L_{mic}(p,t) = \left(\frac{N_{agg/L}(p,t)}{N_{agg/L,RT}} \frac{m_{uni}(p,t)}{m_{ts}(p,t)} + 1 \right) \left(\frac{m_{ts}(p,t) - m_{ts}(0,0)}{m_{ts}(0,0)} + 1 \right) L_{ts}(0,0) \frac{N_{agg/L,RT}}{N_{agg/L}(p,t)} \tag{S8a}$$

which can be rewritten as:

$$L_{mic}(p,t)=\left(\frac{N_{agg/L}(p,t)}{N_{agg/L,RT}}\frac{m_{uni}(p,t)}{m_{ts}(0,0)}+\frac{m_{ts}(p,t)}{m_{ts}(0,0)}\right)L_{ts}(0,0)\frac{N_{agg/L,RT}}{N_{agg/L}(p,t)} \tag{S8b}$$

and:

$$L_{mic}(p,t)=\frac{m_{uni}(p,t)}{m_{ts}(0,0)}L_{ts}(0,0)+\frac{m_{ts}(p,t)}{m_{ts}(0,0)}\frac{N_{agg/L,RT}}{N_{agg/L}(p,t)}L_{ts}(0,0) \tag{S8c}$$

Interestingly, according to equation S7, we have:

$$\frac{m_{ts}(p,t)}{m_{ts}(0,0)}=\frac{m_{uni}(p,0)-m_{uni}(p,t)}{m_{ts}(0,0)}+1 \tag{S9}$$

Therefore:

$$\frac{m_{ts}(p,t)}{m_{ts}(0,0)}\frac{N_{agg/L,RT}}{N_{agg/L}(p,t)}L_{ts}(0,0)=\left(\frac{m_{uni}(p,0)-m_{uni}(p,t)}{m_{ts}(0,0)}+1\right)\frac{N_{agg/L,RT}}{N_{agg/L}(p,t)}L_{ts}(0,0)=L_{ts}(p,t) \tag{S10}$$

Thus equation S8c becomes:

$$L_{mic}(p,t)=\frac{m_{uni}(p,t)}{m_{ts}(0,0)}L_{ts}(0,0)+L_{ts}(p,t) \tag{S11a}$$

Equation S11a can be rewritten as:

$$L_{mic}(p,t)=\frac{m_{uni}(p,0)-\left[m_{uni}(p,0)-m_{uni}(p,t)\right]}{m_{ts}(0,0)}L_{ts}(0,0)+L_{ts}(p,t) \tag{S11b}$$

To further simplify equation S11, we recall that $m_{\text{added}}(p,0) = p\, m_{\text{seeds,RT}}$, and $m_{\text{seeds,RT}} = m_{\text{uni}}(0,0) + m_{\text{ts}}(0,0)$, thus :

$$m_{uni}(p,0)=m_{uni}(0,0)+m_{added}(p,0)=(p+1)m_{seed,RT}-m_{ts}(0,0) \tag{S12}$$

We can then incorporate S12 into S11b:

$$L_{mic}(p,t)=\left\{\frac{(p+1)m_{seed,RT}-m_{ts}(0,0)}{m_{ts}(0,0)}-\frac{\left[m_{uni}(p,0)-m_{uni}(p,t)\right]}{m_{ts}(0,0)}\right\}L_{ts}(0,0)+L_{ts}(p,t) \tag{S13a}$$

S13a can be rearranged to give:

$$L_{mic}(p,t)=\frac{(p+1)m_{seed,RT}}{m_{ts}(0,0)}L_{ts}(0,0)-\left\{\frac{\left[m_{uni}(p,0)-m_{uni}(p,t)\right]}{m_{ts}(0,0)}+1\right\}L_{ts}(0,0)+L_{ts}(p,t) \tag{S13b}$$

From equation S4, we have:

$$\left(\frac{m_{uni}\left(p,0\right)-m_{uni}\left(p,t\right)}{m_{ts}\left(0,0\right)}+1\right)L_{ts}\left(0,0\right)=L_{ts}\left(p,t\right)\frac{N_{agg/L}\left(p,t\right)}{N_{agg/L,RT}} \quad \text{(S14)}$$

Finally by injecting equation S14 into equation S13b, we obtain:

$$L_{mic}\left(p,t\right)=\left(p+1\right)\frac{m_{seed,RT}}{m_{ts}\left(0,0\right)}L_{ts}\left(0,0\right)-L_{ts}\left(p,t\right)\frac{N_{agg/L}\left(p,t\right)}{N_{agg/L,RT}}+L_{ts}\left(p,t\right) \quad \text{(S15)}$$

Equation S15 can be reorganized to give equation (3) main text:

$$L_{mic}\left(p,t\right)=L_{ts}\left(0,0\right)\left[\left(p+1\right)\frac{m_{seeds,RT}}{m_{ts}\left(0,0\right)}\right]+L_{ts}\left(p,t\right)\left[1-\frac{N_{agg/L}\left(p,t\right)}{N_{agg/L,RT}}\right] \quad \text{(3, main text)}$$

Section II: Effect of fragmentation on $L_{mic}(p,t)$ during annealing

When the seed solution was heated at 75 ^{o}C, some of the seeds dissolved, adding unimer to the solution. During annealing time at 75 ^{o}C, some of the surviving seeds fragmented, increasing the number of seeds with time, $N_{ts,f}(p,t)$, and all the surviving seeds grew by seeded growth, decreasing the total mass of unimer present in solution, $m_{uni}(p,t)$. In the control experiments, once the solution was cooled to room temperature, the remaining unimer in solution grew back onto the surviving seeds to give micelles of length $L_{mic}(p,t)$. Since the seed solution we used did not show any sign of fragmentation when aged one year at room temperature, we assume that seed fragmentation only happened when the seeds were annealed at 75 ^{o}C.

If we consider that lateral growth can be neglected, then the linear aggregation number of the micelles is the same at 75 ^{o}C as at room temperature, $N_{agg/L,RT} = N_{agg/L}(p,t)$. We can thus rewrite equation S5 to obtain the length of the micelles regrown at room temperature after being annealed at 75 ^{o}C for a time t:

$$L_{mic}\left(p,t\right)=\left(\frac{m_{uni}\left(p,t\right)}{m_{ts,f}\left(p,t\right)}+1\right)L_{ts,f}\left(p,t\right) \tag{S16}$$

where $m_{ts,f}(p,t)$ is the mass of the fragmented seeds that were annealed for a time t at 75 ^{o}C before the solution was cooled to room temperature. Equation S16 is quite complex since $L_{ts,f}(p,t)$ and $m_{ts,f}(p,t)$ are both impacted by the fragmentation events. Indeed, due to fragmentation equation S4 (taking into account that $N_{agg/L,RT} = N_{agg/L}(p,t)$) is not valid anymore:

$$L_{ts,f}\left(p,t\right)\neq\left(\frac{m_{uni}\left(p,0\right)-m_{uni}\left(p,t\right)}{m_{ts}\left(0,0\right)}+1\right)L_{ts}\left(0,0\right) \tag{S17}$$

To simplify equation S16, we consider the hypothetical case where the seeds fragmented but where no unimer grew on the seeds at 75 ^{o}C. In such case, some seeds dissolved as soon as the solution temperature reached 75 ^{o}C, and the remaining seeds fragmented with time without further dissolution, but without growing either, *i.e.*, $m_{uni}(p,t) = m_{uni}(p,0)$. In this hypothetical case, the unimer only grew onto the seeds once the solution reached room temperature. Equation S16 can thus be rewritten as a function of the mass of fragmented seeds, $m_{ts,f}(p,t)$, the length of the fragmented seeds, $L_{ts,f}(p,t)$, and the mass of unimer in solution just after it reached 75 ^{o}C, $m_{uni}(p,0)$:

$$L_{mic}\left(p,t\right)=\left(\frac{m_{uni}\left(p,0\right)}{m_{ts,f}\left(p,t\right)}+1\right)L_{ts,f}\left(p,t\right) \tag{S18}$$

with $m_{uni}(p,0)$ defined in equation S12. Since in this specific case, the micelles only regrew when the solution was cooled to room temperature, we can write that $m_{ts,f}(p,t)$ = $m_{ts}(0,0)$ which corresponds to the mass of seeds that were still present in solution when the annealing temperature was reached.

From equation S12, we have:

$$L_{mic}\left(p,t\right)=\left(\frac{m_{uni}\left(0,0\right)+m_{added}\left(p,0\right)}{m_{ts}\left(0,0\right)}+1\right)L_{ts,f}\left(p,t\right) \tag{S19}$$

Equation S12 can also be rearranged to give:

$$\frac{m_{uni}\left(0,0\right)+m_{added}\left(p,0\right)}{m_{ts}\left(0,0\right)}=\frac{\left(p+1\right)m_{seed,RT}-m_{ts}\left(0,0\right)}{m_{ts}\left(0,0\right)} \tag{S20}$$

Then equation S19 can be written as equation 7 main text.

$$L_{mic}\left(p,t\right)=\left(p+1\right)\frac{m_{seed,RT}}{m_{ts}\left(0,0\right)}L_{ts,f}\left(p,t\right) \tag{7, main text}$$

At this stage, we have considered that the unimer was growing back onto the seeds at room temperature only. Figure 2c,d (main text) shows, however, that seeds grew during annealing. In the case where $N_{agg/L,RT}$ = $N_{agg/L}(p,t)$, the temperature at which the seeds grew does not affect their final lengths, *i.e.*, $L_{mic}(p,t)$. $L_{mic}(p,t)$, however, would be changed if seed fragmentation varied as a function of the amount of unimer added to the solution. To quantify the effect of the amount of unimer on seed fragmentation, we define a function $\delta(p,t)$. $\delta(p,t)$ is such that $N_{ts,f}(p,t) = \delta(p,t)\ N_{ts,f}(0,t)$. $\delta(p,t) > 1$ means that the number of fragmented seeds increases in presence of added unimer, in other words, fragmentation is promoted by the addition of PFS_{53}-*b*-PI_{637} unimer (since more seeds are created). $\delta(p,t) < 1$ indicates the number of fragmented seeds decreases in presence of extra unimer, this would be the case if the unimer favors fusion between seeds. $\delta(p,t) = 1$ means that the addition of PFS_{53}-*b*-PI_{637} unimer does not affect the number of surviving seeds.

The lengths of the seeds would also be affected by fragmentation or fusion. For example if two seeds fuse, the length of the resulting seed would double, and if all the seeds fragment in

two, the average length obtained from the resulting seeds would be half the average length of the non-fragmented seeds. In other words, $L_{ts,f}(p,t) = (1/\delta(p,t))\ L_{ts,f}(0,t)$. Since the mass of seeds without considering their growth, $m_{ts,f}(p,t)$, is proportional to the product $N_{ts,f}(p,t) \times L_{ts,f}(p,t)$, we can conclude that $m_{ts,f}(p,t)$ remains constant for a given value of p. As a consequence, considering that $L_{ts,f}(p,t) = (1/\delta(p,t))\ L_{ts,f}(0,t)$ equation 7, main text becomes:

$$L_{mic}(p,t) = (1+p)\frac{m_{seed,RT}}{m_{ts}(0,0)}\frac{L_{ts,f}(0,t)}{\delta(p,t)} \tag{S21}$$

Using equation 4, main text, we can rewrite equation S21:

$$L_{mic}(p,t) = \frac{L_{mic}(0,0)}{L_{ts}(0,0)}(1+p)\frac{L_{ts,f}(0,t)}{\delta(p,t)} \tag{S22}$$

Then, from equation 8, main text, we deduce:

$$L_{mic}(p,t) = (1+p)\frac{L_{mic}(0,t)}{\delta(p,t)} \tag{S23}$$

The function $\delta(p,t)$ can be obtained from the slope of the plot $L_{mic}(p,t)$ as a function of $[L_{mic}(0,t)\times(1+p)]$ for each annealing time studied. In Supplementary Fig. 19, we show the plot of $L_{mic}(p,t)$ as a function of $[L_{mic}(100,t)\times(1+p)]$ (considering that $L_{mic}(0,t) \approx L_{mic}(100,t)$). From this slope, we deduce that $\delta(p,t) = 1$, *i.e*, $N_{ts}(p,t) = N_{ts}(0,t)$, and equation S23 becomes:

$$L_{mic}(p,t) = (1+p)L_{mic}(0,t) \tag{S24}$$

We can thus conclude that the addition of PFS_{53}-*b*-PI_{637} unimer in the solution did not affect the number of surviving seeds: $N_{ts,f}(p,t) = N_{ts,f}(0,t)$.

Section III: Growth kinetics at 75 °C

In this section we derive the equations governing the micelle growth kinetics at 75 °C. We first summarize the events that take place at the annealing temperature. When a seed solution is heated to 75 °C, some unimer are transferred from the seeds to the surrounding solution following two different pathways: 1) seed shortening, where the seeds partially dissolve to reach the critical length that we observed in the dissolution experiments, and 2) seed dissolution, where entire seeds dissolve. Experimentally, we observe that micelle elongation at 75 °C occurs on a much longer time scale than seed dissolution. Therefore we can assume that the contribution of seed dissolution to the unimer concentration at 75 °C is "instantaneous", i.e. prior to the onset of micelle growth.

When no extra unimer is added to the solution, seed growth at 75 °C can be followed as a function of time :

$$L_{ts}\left(p,t\right)=L_{ts}\left(p,0\right)\left[1+\frac{m_{uni}\left(p,0\right)-m_{uni}\left(p,t\right)}{m_{ts}\left(p,0\right)}\right] \tag{S25}$$

where $L_{ts}(p,0)$ is the length of the starting seeds. We want to remind the reader that $L_{ts}(p,0) = L_{ts}(0,0)$, and $m_{ts}(p,0) = m_{ts}(0,0)$. $m_{uni}(p,t)$ is related to the number concentration of unimer, $[N_{uni}(p,t)]$ present in solution at 75°C at an annealing time, t:

$$m_{uni}\left(p,t\right)=\left[N_{uni}\left(p,t\right)\right]V_{sol}\,M_{o} \tag{S26}$$

Eq. S25 thus becomes:

$$L_{ts}\left(0,t\right)=L_{ts}\left(0,0\right)\left[1+\frac{\left(\left[N_{uni}\left(p,0\right)\right]-\left[N_{uni}\left(p,t\right)\right]\right)V_{sol}\,M_{0}}{m_{ts}\left(0,0\right)}\right] \tag{S27}$$

When a unimer adds to a seed, this unimer is removed from the solution. Under these conditions, micelle growth is governed by the second-order rate expression

$$\frac{d\left[N_{uni}(p,t)\right]}{dt}=-k_2\left[N_{ts}(0,t)\right]\left[N_{uni}(p,t)\right] \tag{S28a}$$

where $[N_{ts}(0,t)]$ is the number concentration of seeds in solution at 75 ^{o}C, and k_2 is the characteristic rate constant for block copolymer deposition on the open end of a seed or micelle. Since the number of seeds is constant in the absence of fragmentation, micelle growth kinetics can be described by pseudo-first-order kinetics.

$$\frac{d\left[N_{uni}(p,t)\right]}{dt} = -k'\left[N_{uni}(p,t)\right] \tag{S28b}$$

and k' is the pseudo first-order rate constant for the addition of the BCP chains. These expressions assume that there is only one population of unimer in solution and that its addition to the seeds to the seeds is governed by a unique rate constant.

The solution of this equation is:

$$\left[N_{uni}(p,t)\right] = \left[N_{uni}(p,0)\right]\, e^{-k't} \tag{S29}$$

Incorporating equation S29 in equation S27 and taking into account equation S28a,b, we obtain:

$$L_{ts}\left(p,t\right) = L_{ts}\left(0,0\right)\left[1+\frac{m_{uni}\left(p,0\right)}{m_{ts}\left(0,0\right)}\left(1-e^{-k't}\right)\right] \tag{S30}$$

We recall that:

$$\frac{m_{uni}\left(p,0\right)}{m_{ts}\left(0,0\right)} = \frac{m_{seed,RT} - m_{ts}\left(0,0\right) + p\, m_{seed,RT}}{m_{ts}\left(0,0\right)} = \left(p+1\right)\frac{m_{seed,RT}}{m_{ts}\left(0,0\right)} - 1 \tag{S31a}$$

and that:

$$\frac{m_{seed,RT}}{m_{ts}\left(0,0\right)} = \frac{L_{mic}\left(0,0\right)}{L_{ts}\left(0,0\right)} \tag{S31b}$$

We can thus rewrite equation S30 as a function of $L_{mic}(0,0)$ and $L_{ts}(0,0)$:

$$L_{ts}(0,t) = L_{ts}(0,0)\left[1+\left(\frac{L_{mic}\left(0,0\right)}{L_{ts}\left(0,0\right)}\left(1+p\right)-1\right)\left(1-e^{-k't}\right)\right] \tag{S32}$$

Equation S32 predicts an exponential growth of micelle length over time, as observed in the simulations of Hu and coworkers [3]. Their simulations involved assumptions consistent with those made in this section.

Boott et al. [4], however, have shown that the growth rates are highly sensitive to differences in the length of the crystalline block. Thus, we should consider the effect of block copolymer molecular weight distribution on the micelle growth kinetics. Block copolymers are not monodisperse and contain a distribution of chains with different block ratios, ρ_s. If the block ratio or the lengths of the crystalline block affect the addition rate, then each population would add to the end of a seed with a different rate constant, k_s. Under these circumstances, equation S29 becomes:

$$\left[N_{uni}(p,t)\right] = \sum\left[N_{uni}(p,t)\right]_s = \left[N_{uni}(p,0)\right]\sum P_s\, e^{-k_s t} \tag{S33}$$

where $[N_{uni}(p,t)]$ is the concentration of the unimer of block ratio ρ_s. P_s is the number of block copolymer chains of block ratio ρ_s, normalized by the total amount of unimer. Replacing the sum by an integral leads to:

$$\left[N_{uni}(p,t)\right] = \left[N_{uni}(p,0)\right]\int_0^\infty P(s)\, e^{-k^* s t} ds \tag{S34}$$

where k^* is the characteristic rate constant with $k_s = k^* \times s$. Johnston [5] has shown that the form of equation S34 can be described by a stretched exponential function:

$$\left[N_{uni}(p,t)\right] = \left[N_{uni}(p,0)\right]\int_0^\infty P(s)\, e^{-k^* s t} ds = \left[N_{uni}(p,0)\right] e^{-\left(k^* t\right)^\beta} \tag{S35}$$

where β is the stretching exponent. Equation S32 thus becomes:

$$L_{ts}(p,t) = L_{ts}(0,0)\left[1 + \left(\frac{L_{mic}(0,0)}{L_{ts}(0,0)}(1+p) - 1\right)\left(1 - e^{-\left(k^* t\right)^\beta}\right)\right] \tag{S36}$$

Equation S36 provides a rationale for the finding in Fig. 6 (main text) that the growth kinetics at 75 °C is inconsistent with an exponential growth model and can be fitted to a stretched exponential form.

Supplementary Tables

Table S1: Values of $L_{mic}(p,t)$ of the PFS_{53}-*b*-PI_{637} crystallites heated for different annealing times at 75 ^oC and cooled to room temperature (control experiments).

	100 min	420 min	1200 min	2640 min
$L_{mic}(0,t)$ (nm)	64.4	63.2	59.1	55.4
SEM [a] (%)	5.7	5.0	6.4	8.6
$L_{mic}(0.9,t)$ (nm)	123	113	108	105
SEM [a] (%)	4.0	3.7	4.0	4.5
$L_{mic}(2,t)$ (nm)	187	184	177	161
SEM [a] (%)	3.4	3.6	4.3	3.2
$L_{mic}(3.1,t)$ (nm)	268	255	241	220
SEM [a] (%)	2.7	3.0	3.9	4.1
$L_{mic}(4.5,t)$ (nm)	360	352	327	296
SEM [a] (%)	3.1	2.9	3.0	3.5
$L_{mic}(4.9,t)$ (nm)	384	369	352	323
SEM [a] (%)	2.5	2.4	3.2	3.4

a. SEM = standard error of the mean

Table S2: Values of $L_{ts}(p,t)$ of the PFS_{53}-*b*-PI_{637} crystallites heated for different annealing time at 75 ^{o}C (seed trapping experiments).

	100 min	420 min	1200 min	2640 min
$L_{ts}(0,t)$ (nm)	37.5	35.9	40.5	40.4
SEM [a] (%)	5.7	8.1	7.6	7.5
$L_{ts}(0.9,t)$ (nm)	40.2	44.3	47.7	54.7
SEM [a] (%)	8.8	8.1	6.1	7.2
$L_{ts}(2,t)$ (nm)	44	51.1	56.7	64.6
SEM [a] (%)	7.2	6.5	6.2	7.2
$L_{ts}(3.1,t)$ (nm)	47.6	54.3	63.5	68.7
SEM [a] (%)	5.8	5.8	6.0	6.3
$L_{ts}(4.5,t)$ (nm)	47.5	64.1	70.5	82.2
SEM [a] (%)	6.7	6.8	6.2	5.4
$L_{ts}(4.9,t)$ (nm)	53.4	63.9	72.6	85.9
SEM [a] (%)	6.5	6.9	5.8	6.1

a. SEM = standard error of the mean

Supplementary Figures

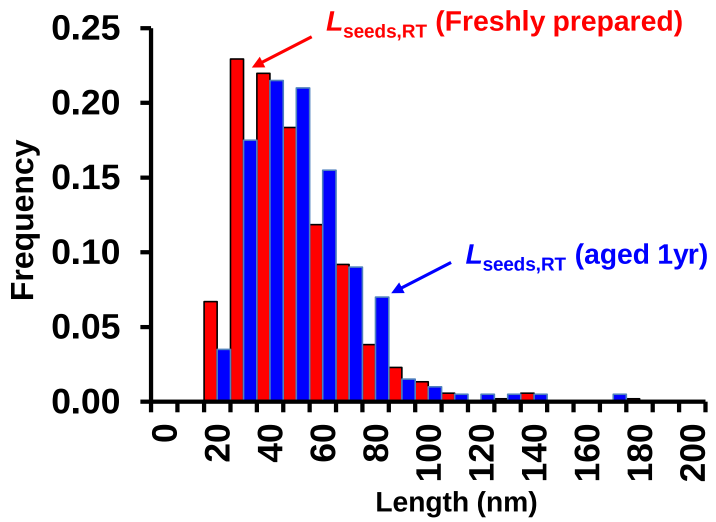

Figure S1: Effect of PFS$_{53}$-*b*-PI$_{637}$ seed crystallite history on their stability at RT. Red histogram, a solution in decane of freshly prepared PFS$_{53}$-*b*-PI$_{637}$ seeds. Blue histogram, a sample of the same seed solution that was allowed to age for 1 yr at room temperature in a sealed vial. The number average length of the freshly prepared seeds crystallites was $L_{seeds,RT}$ = 43.5 nm, similar to that of the sample aged for 1 yr.

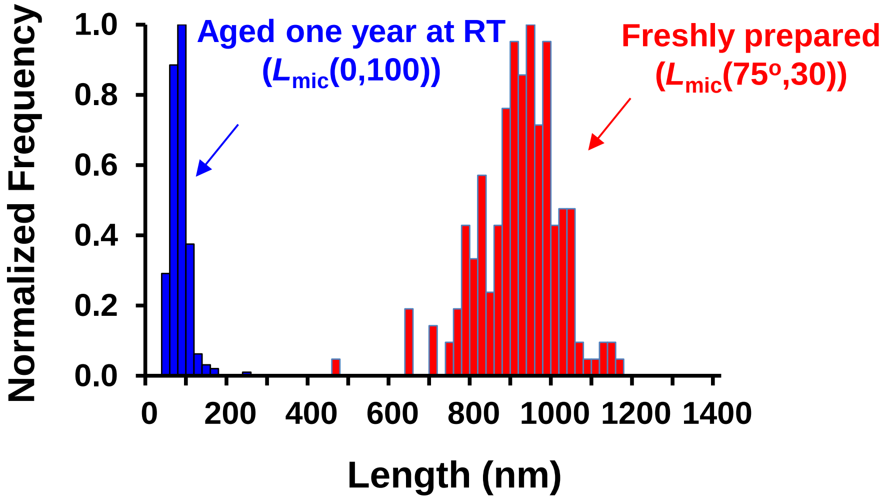

Figure S2: Effect of PFS$_{53}$-*b*-PI$_{637}$ seed crystallite history on their stability against dissolution upon heating. Red histogram, a solution in decane of freshly prepared PFS$_{53}$-*b*-PI$_{637}$ seeds heated for 30 min at 75 °C and cooled to RT. Blue histogram, a sample of the same seed solution that was allowed to age for 1 yr at room temperature in a sealed vial. This solution was then heated for 100 min at 75 °C and cooled to 23 °C. The number average length of the seeds regrown from the solution aged for 1 yr was much smaller (L_{mic}(0,100) = 64 nm) than that of the freshly prepared seeds (L_{mic}(75°,30) = 902 nm). This difference indicates that a much smaller fraction of the aged seeds dissolved upon heating to 75 °C, consistent with a substantial enhancement of the robustness of the seeds due to an increase in crystallinity.

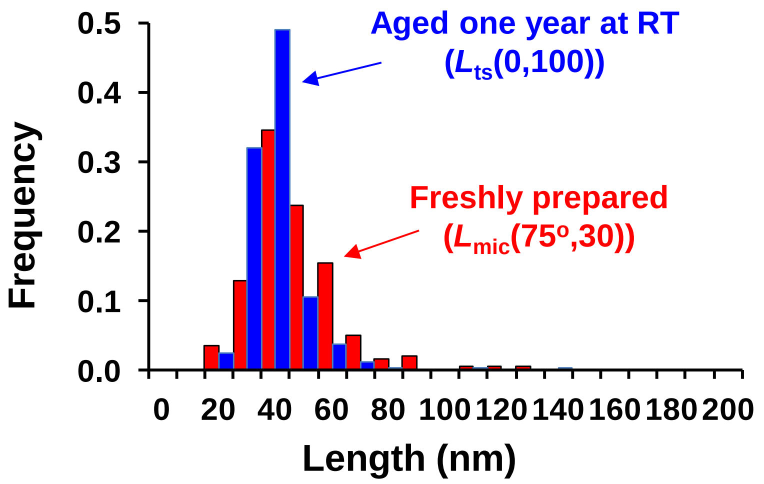

Figure S3: Comparison of the histograms of the length distribution PFS$_{53}$-*b*-PI$_{637}$ seed crystallites that were trapped with PFS$_{60}$-*b*-PDMS$_{660}$ unimer after being heated at 75 °C. Red bars: freshly prepared seed crystallites heated for 30 min and then cooled to RT; blue bars, the same sample aged one year at RT in a sealed vial and heated for 100 min and then cooled to RT. This comparison tells us that in spite of the enhanced crystallinity of the seed solution aged for 1 yr, the length distribution of the surviving seeds following annealing at 75 °C remains essentially unchanged.

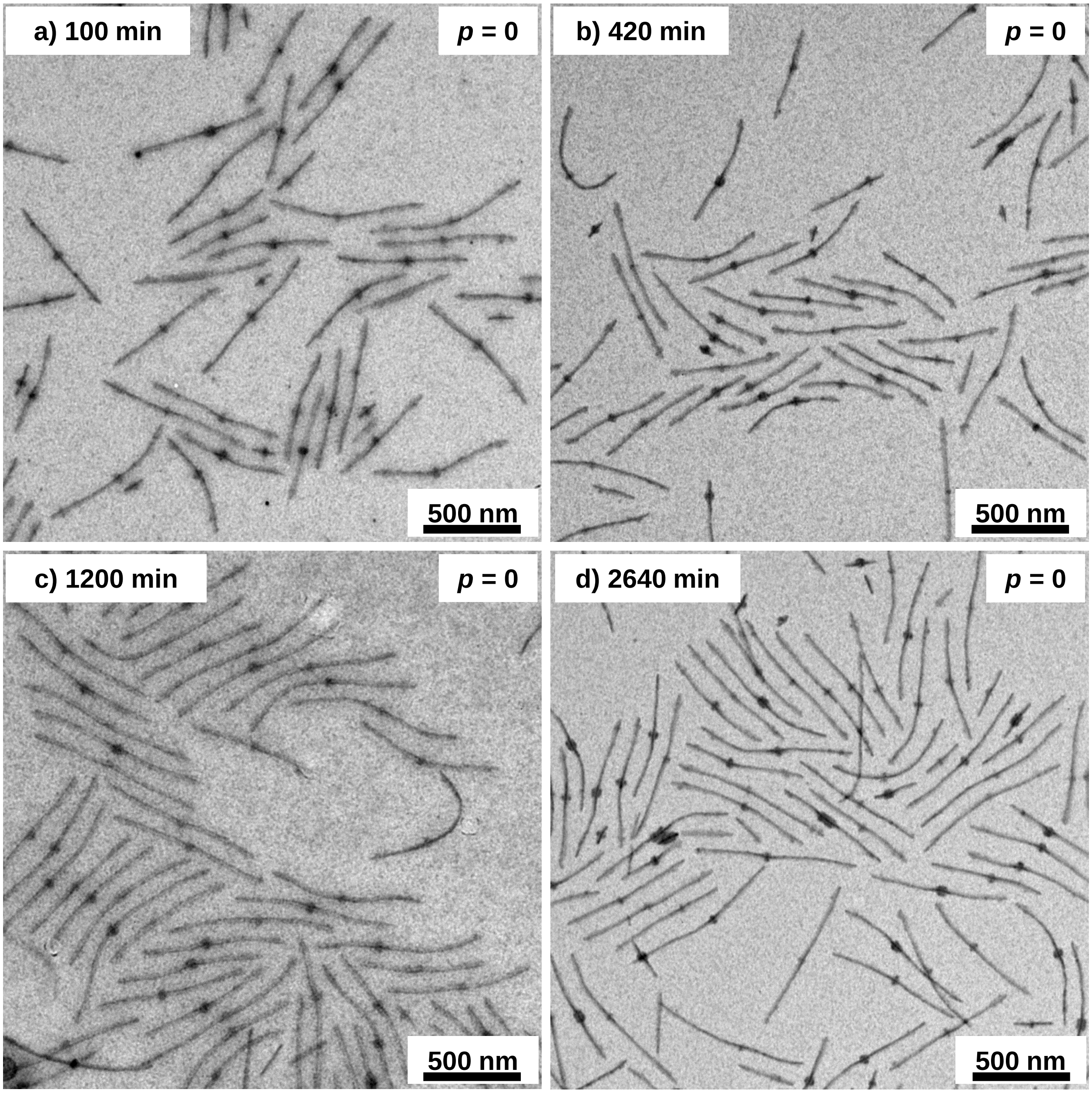

Figure S4: TEM micrographs of PFS_{53}-*b*-PI_{637} seeds trapped after a) 100 min, b) 420 min, c) 1200 min and d) 2640 min of annealing at 75 ^{o}C without PFS_{53}-*b*-PI_{637} unimer added (p = 0).

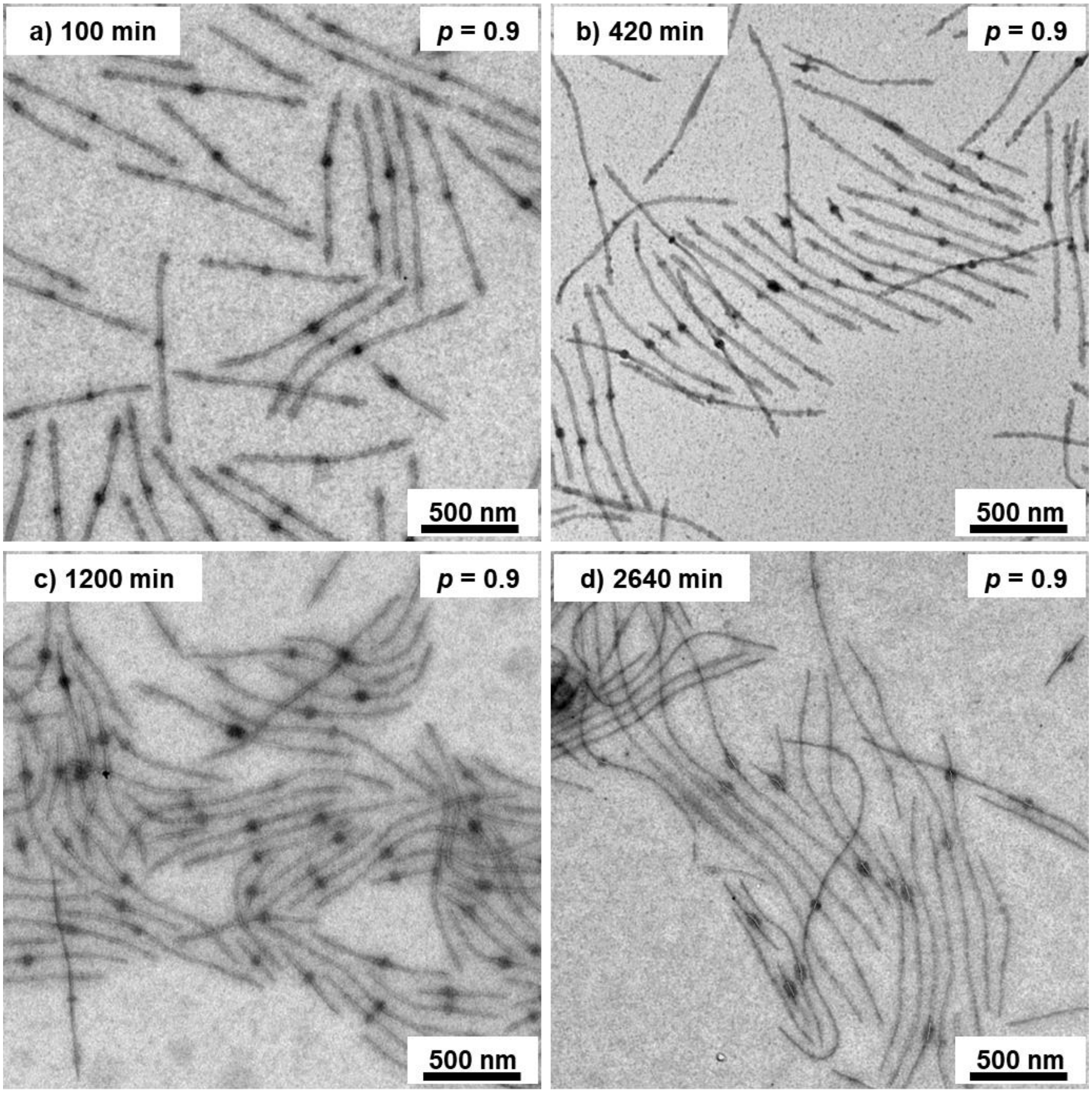

Figure S5: TEM micrographs of PFS_{53}-*b*-PI_{637} seeds trapped after a) 100 min, b) 420 min, c) 1200 min and d) 2640 min of annealing at 75 ^{o}C in the presence of an initial mass ratio of PFS_{53}-*b*-PI_{637} unimer added to PFS_{53}-*b*-PI_{637} seeds p = 0.9.

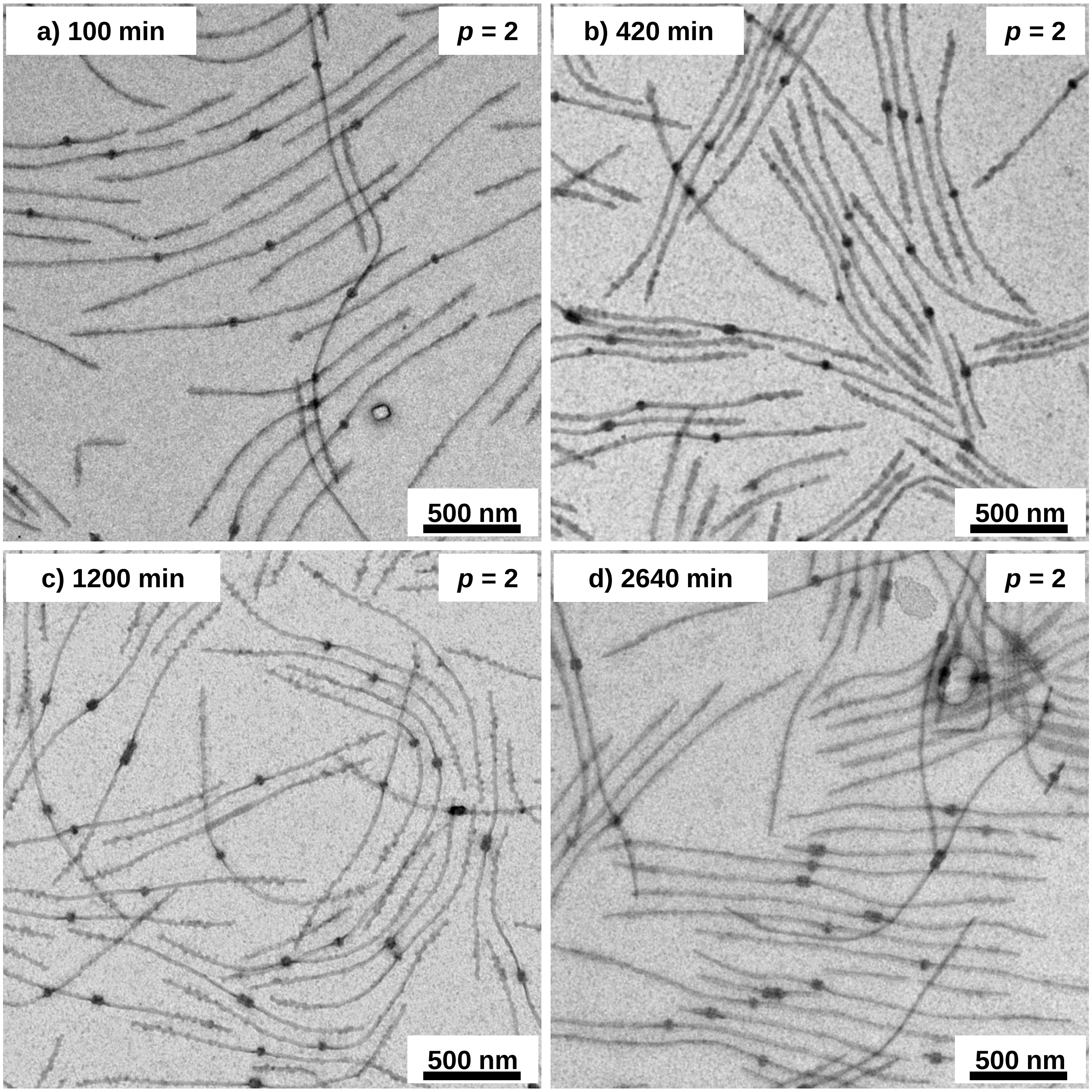

Figure S6: TEM micrographs of PFS_{53}-*b*-PI_{637} seeds trapped after a) 100 min, b) 420 min, c) 1200 min and d) 2640 min of annealing at 75 °C in the presence of an initial mass ratio of PFS_{53}-*b*-PI_{637} unimer added to PFS_{53}-*b*-PI_{637} seeds p = 2.

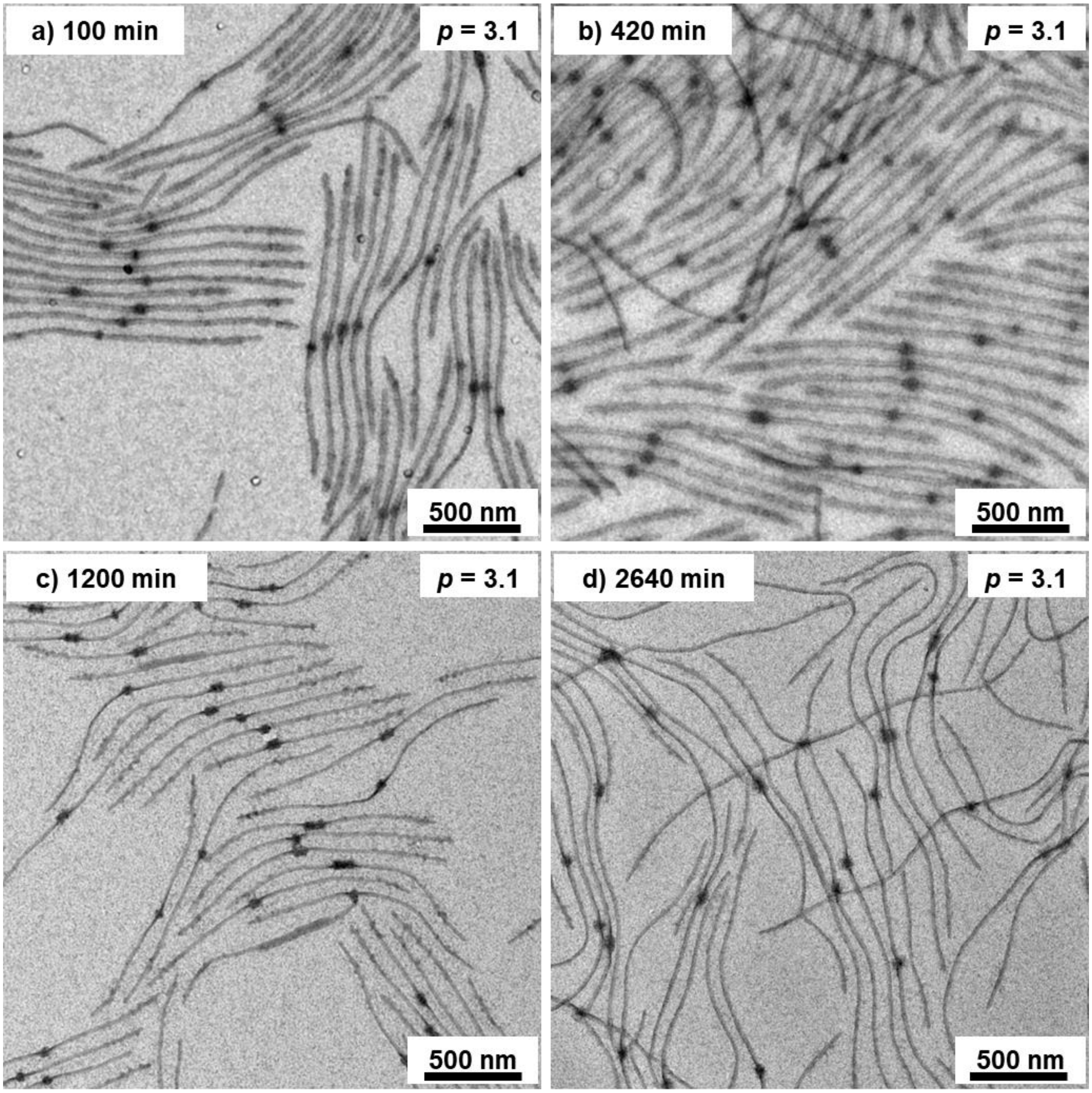

Figure S7: TEM micrographs of PFS_{53}-*b*-PI_{637} seeds trapped after a) 100 min, b) 420 min, c) 1200 min and d) 2640 min of annealing at 75 ^{o}C in the presence of an initial mass ratio of PFS_{53}-*b*-PI_{637} unimer added to PFS_{53}-*b*-PI_{637} seeds p = 3.1.

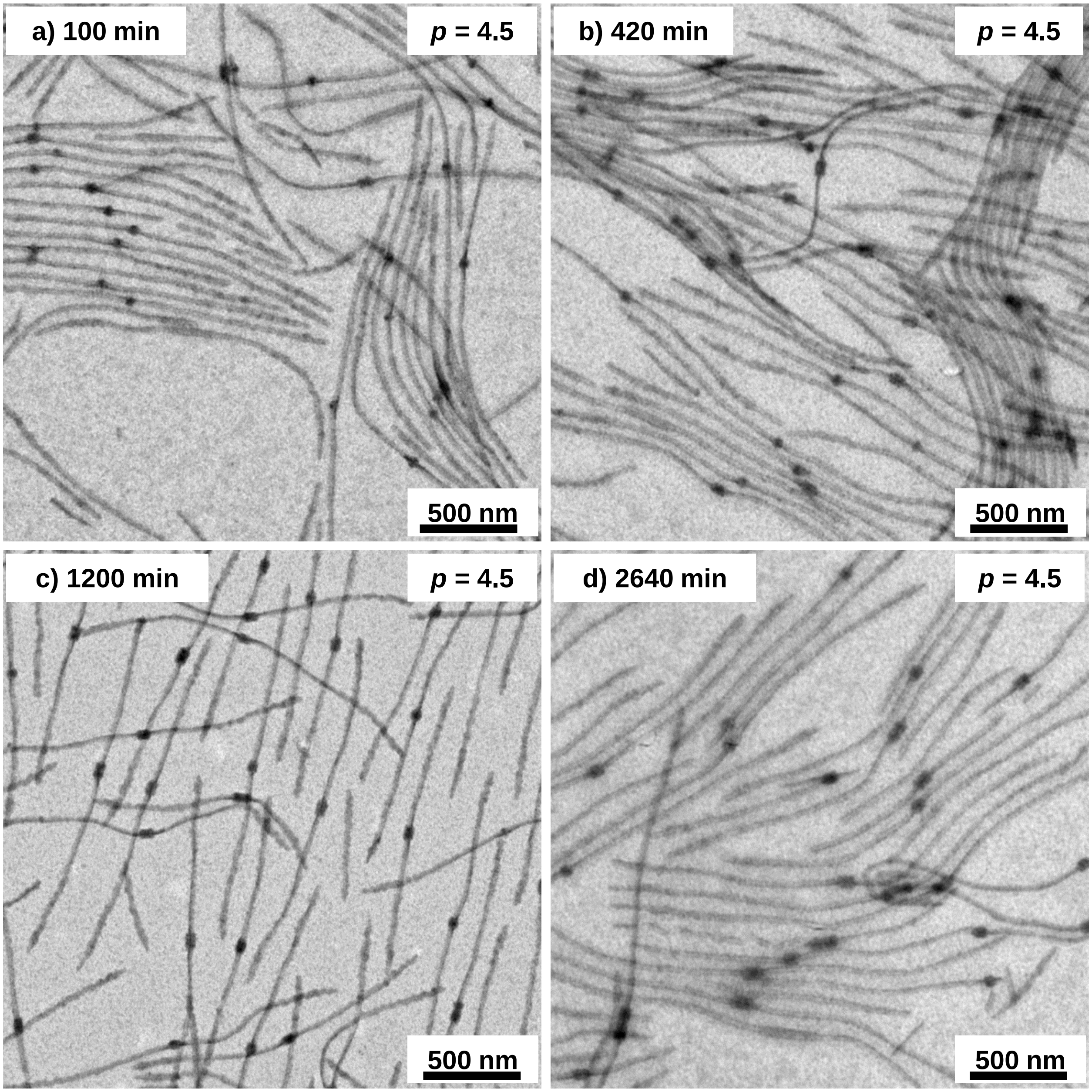

Figure S8: TEM micrographs of PFS_{53}-*b*-PI_{637} seeds trapped after a) 100 min, b) 420 min, c) 1200 min and d) 2640 min of annealing at 75 ^{o}C in the presence of an initial mass ratio of PFS_{53}-*b*-PI_{637} unimer added to PFS_{53}-*b*-PI_{637} seeds p = 4.5.

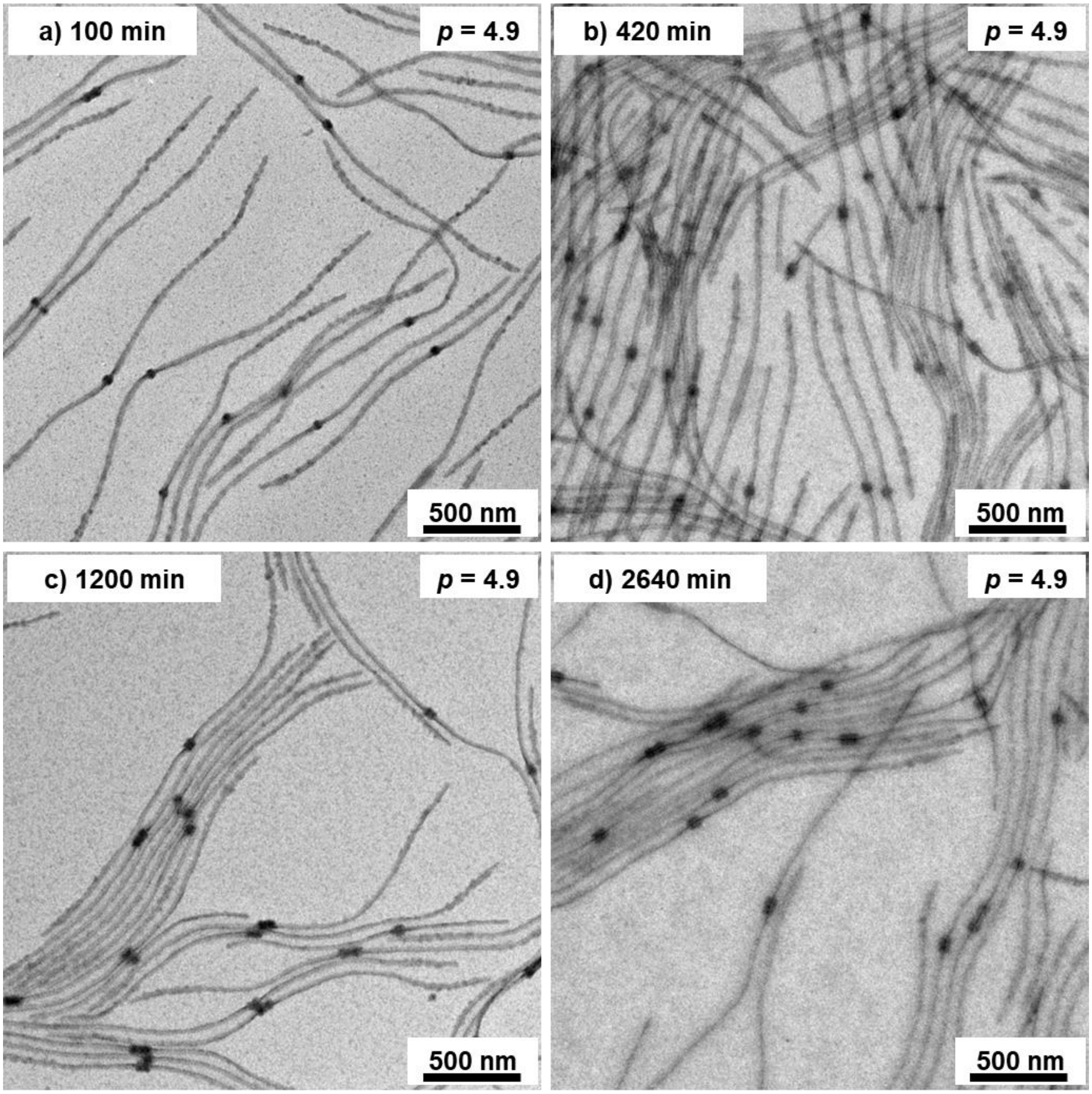

Figure S9: TEM micrographs of PFS_{53}-*b*-PI_{637} seeds trapped after a) 100 min, b) 420 min, c) 1200 min and d) 2640 min of annealing at 75 °C in the presence of an initial mass ratio of PFS_{53}-*b*-PI_{637} unimer added to PFS_{53}-*b*-PI_{637} seeds $p = 4.9$.

***p* = 0**

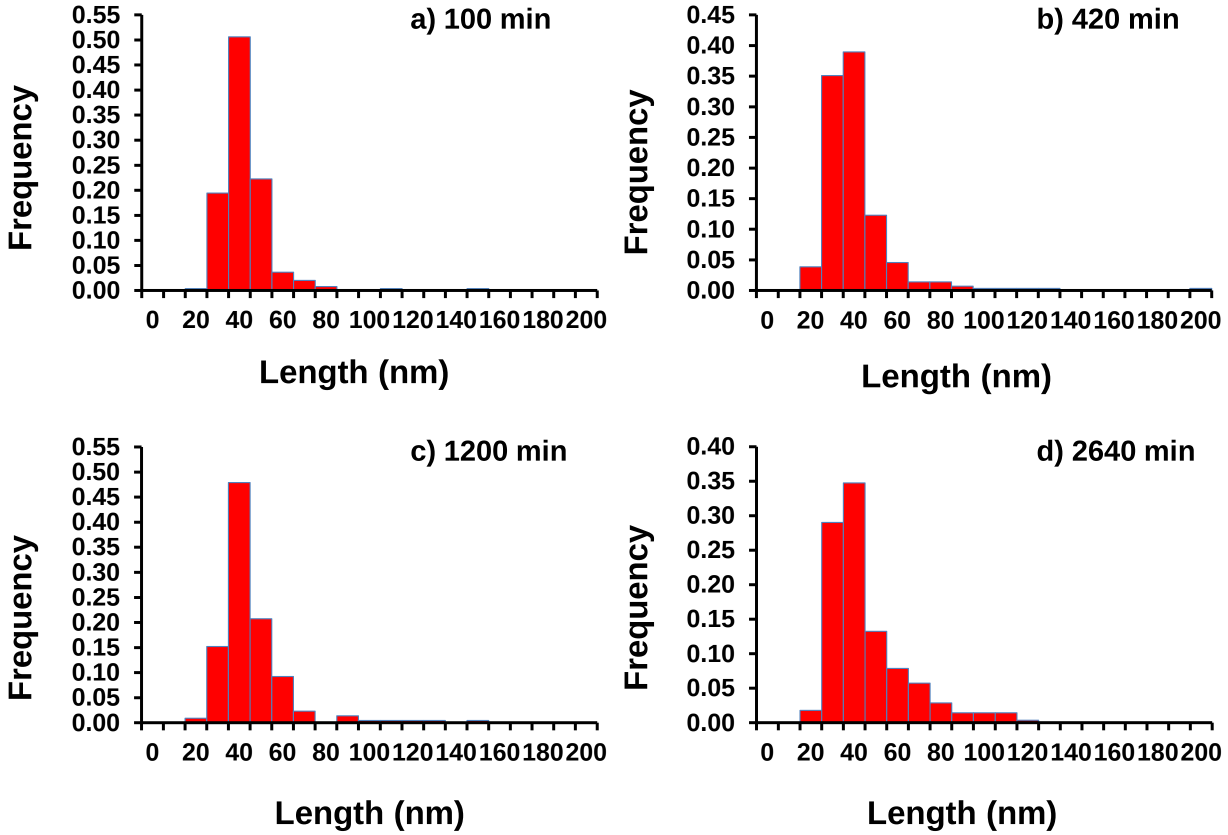

Figure S10: Histograms of the length distributions of PFS_{53}-*b*-PI_{637} trapped seeds after a) 100 min, b) 420 min, c) 1200 min and d) 2640 min of annealing at 75 °C without PFS_{53}-*b*-PI_{637} unimer added (*p* = 0).

***p* = 0.9**

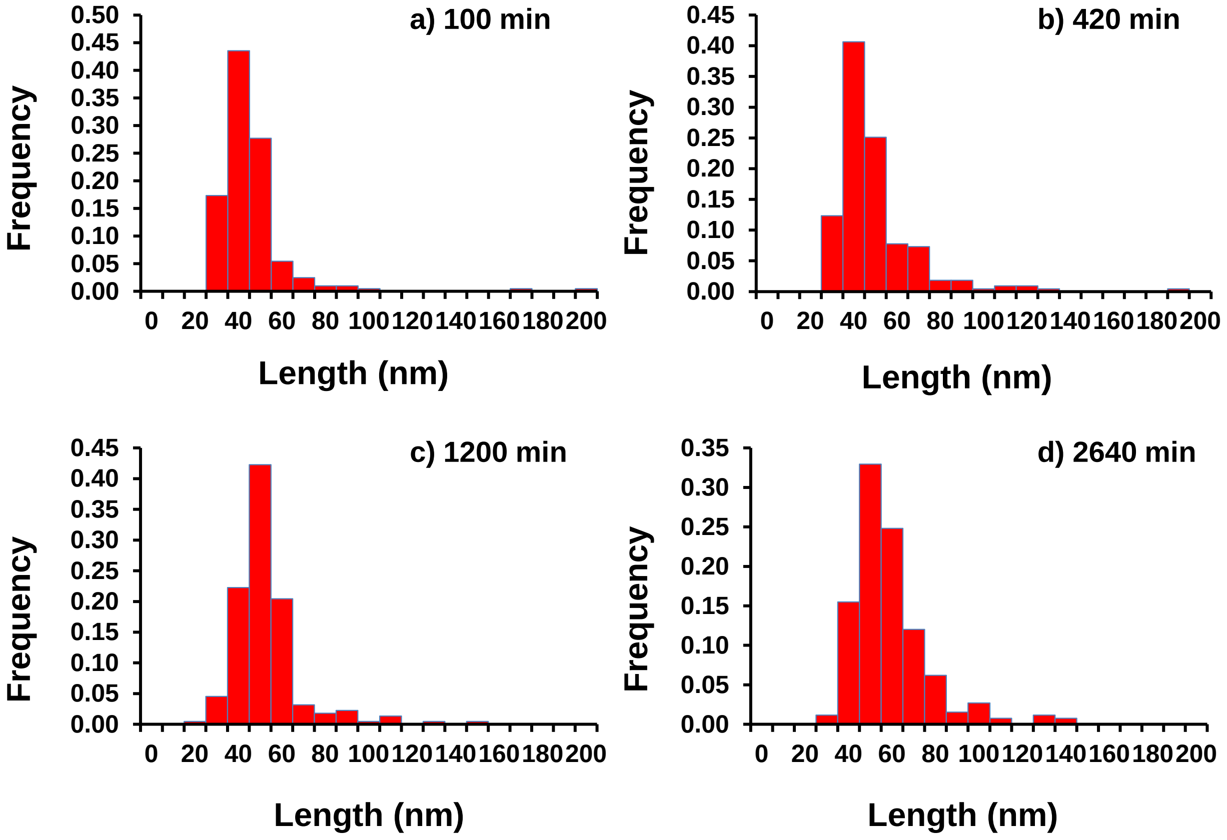

Figure S11: Histograms of the length distributions of PFS$_{53}$-*b*-PI$_{637}$ trapped seeds after a) 100 min, b) 420 min, c) 1200 min and d) 2640 min of annealing at 75 ^{o}C in the presence of an initial mass ratio of PFS$_{53}$-*b*-PI$_{637}$ unimer added to PFS$_{53}$-*b*-PI$_{637}$ seeds *p* = 1.

***p* = 2**

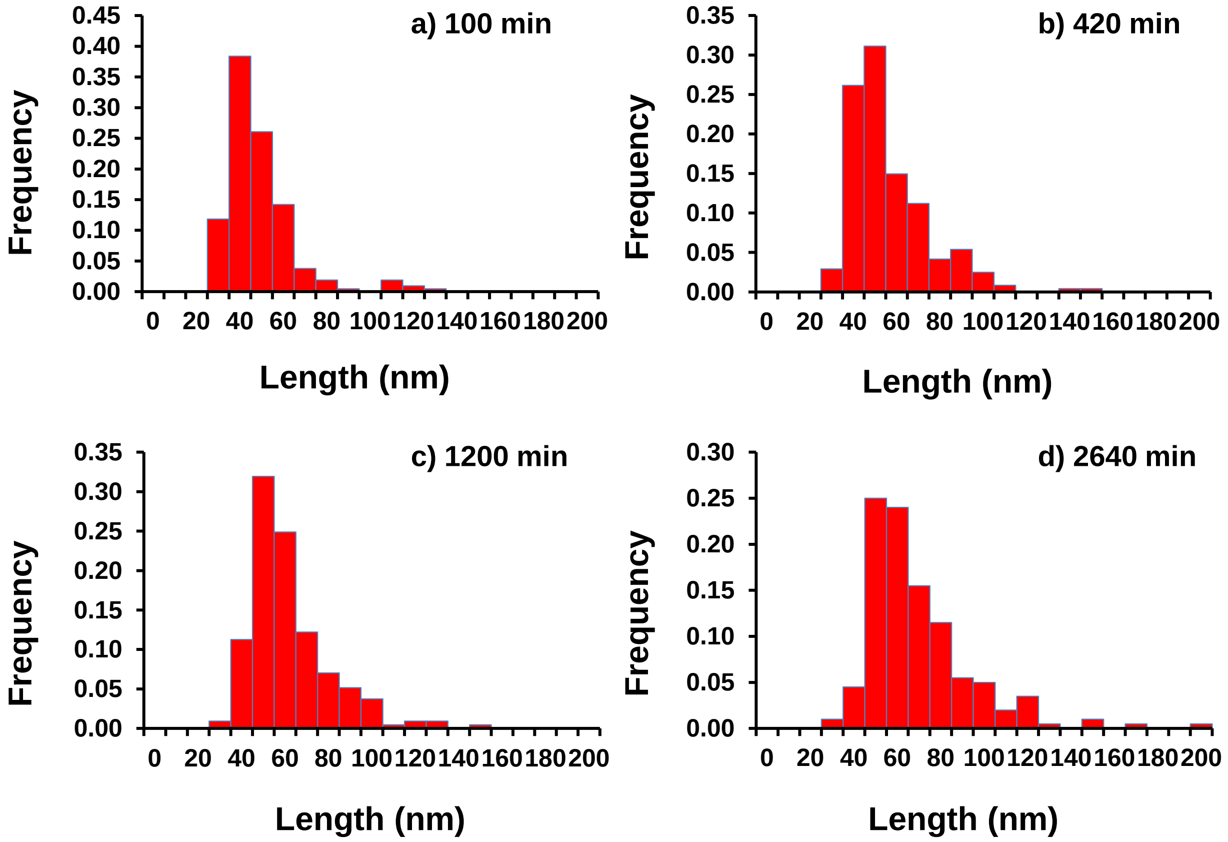

Figure S12: Histograms of the length distributions of PFS_{53}-*b*-PI_{637} trapped seeds after a) 100 min, b) 420 min, c) 1200 min and d) 2640 min of annealing at 75 ^{o}C in the presence of an initial mass ratio of PFS_{53}-*b*-PI_{637} unimer added to PFS_{53}-*b*-PI_{637} seeds $p = 2$.

***p* = 3.1**

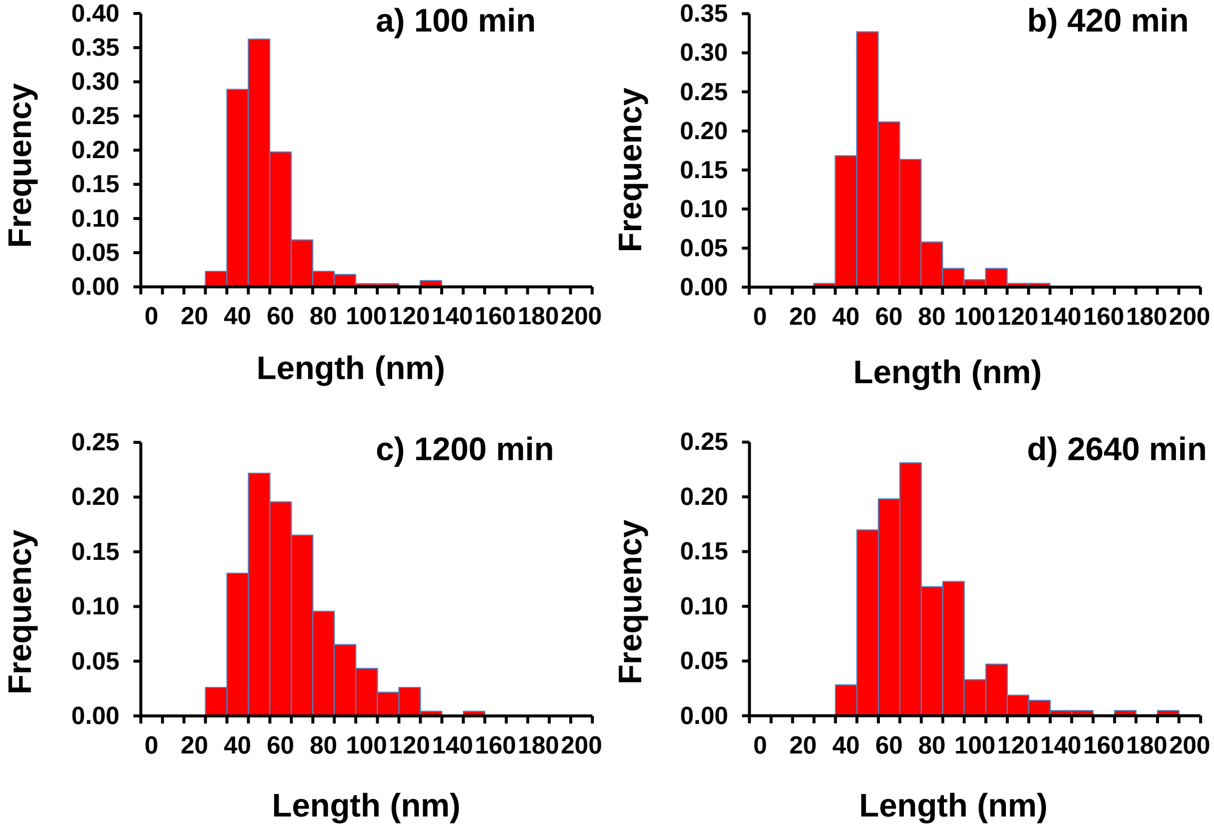

Figure S13: Histograms of the length distributions of PFS_{53}-*b*-PI_{637} trapped seeds after a) 100 min, b) 420 min, c) 1200 min and d) 2640 min of annealing at 75 °C in the presence of an initial mass ratio of PFS_{53}-*b*-PI_{637} unimer added to PFS_{53}-*b*-PI_{637} seeds *p* = 3.

***p* = 4.5**

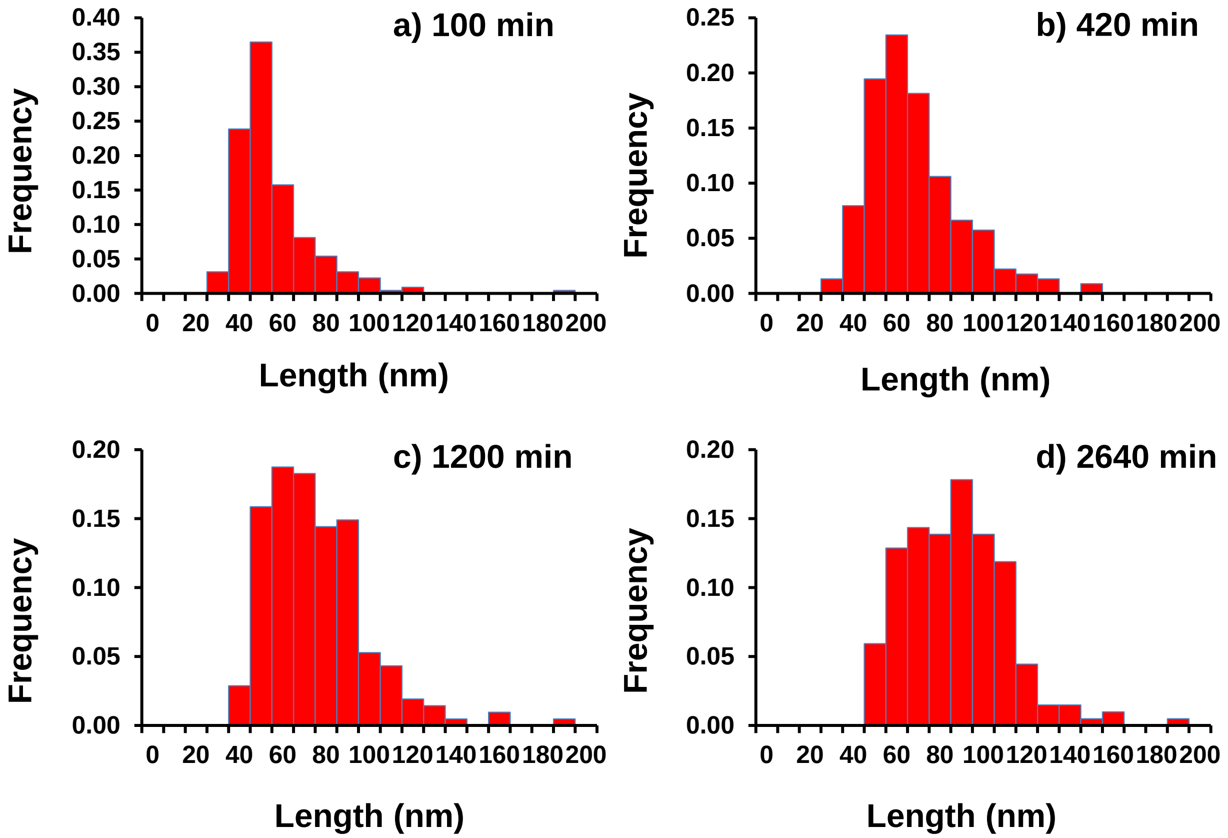

Figure S14: Histograms of the length distributions of PFS_{53}-*b*-PI_{637} trapped seeds after a) 100 min, b) 420 min, c) 1200 min and d) 2640 min of annealing at 75 ^{o}C in the presence of an initial mass ratio of PFS_{53}-*b*-PI_{637} unimer added to PFS_{53}-*b*-PI_{637} seeds *p* = 4.5.

***p* = 4.9**

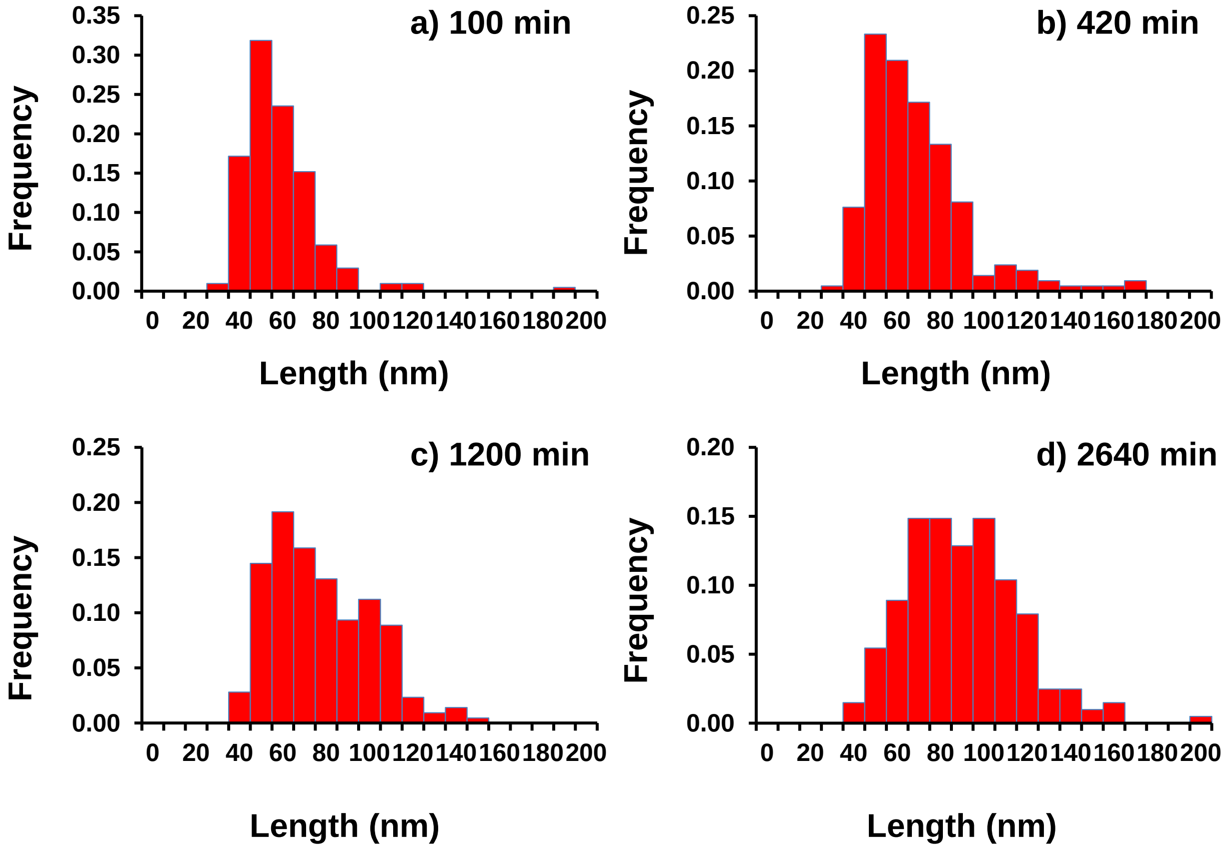

Figure S15: Histograms of the length distributions of PFS_{53}-*b*-PI_{637} trapped seeds after a) 100 min, b) 420 min, c) 1200 min and d) 2640 min of annealing at 75 ^oC in the presence of an initial mass ratio of PFS_{53}-*b*-PI_{637} unimer added to PFS_{53}-*b*-PI_{637} seeds p = 4.8.

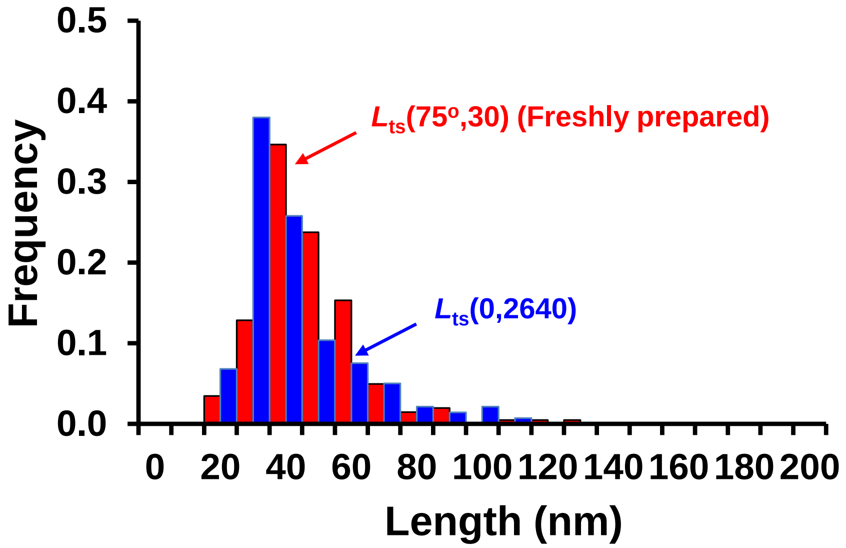

Figure S16: Histograms of the length of PFS_{53}-*b*-PI_{637} seed crystallites in decane that were trapped with added PFS_{60}-*b*-$PDMS_{660}$ unimer after being heated at 75 ^{o}C, then cooled to RT and stained with Karstedt's catalyst. Red bars: a sample of freshly prepared seeds in decane heated for 30 min; blue bars: the 1 yr aged sample heated for 2640 min at 75 ^{o}C.

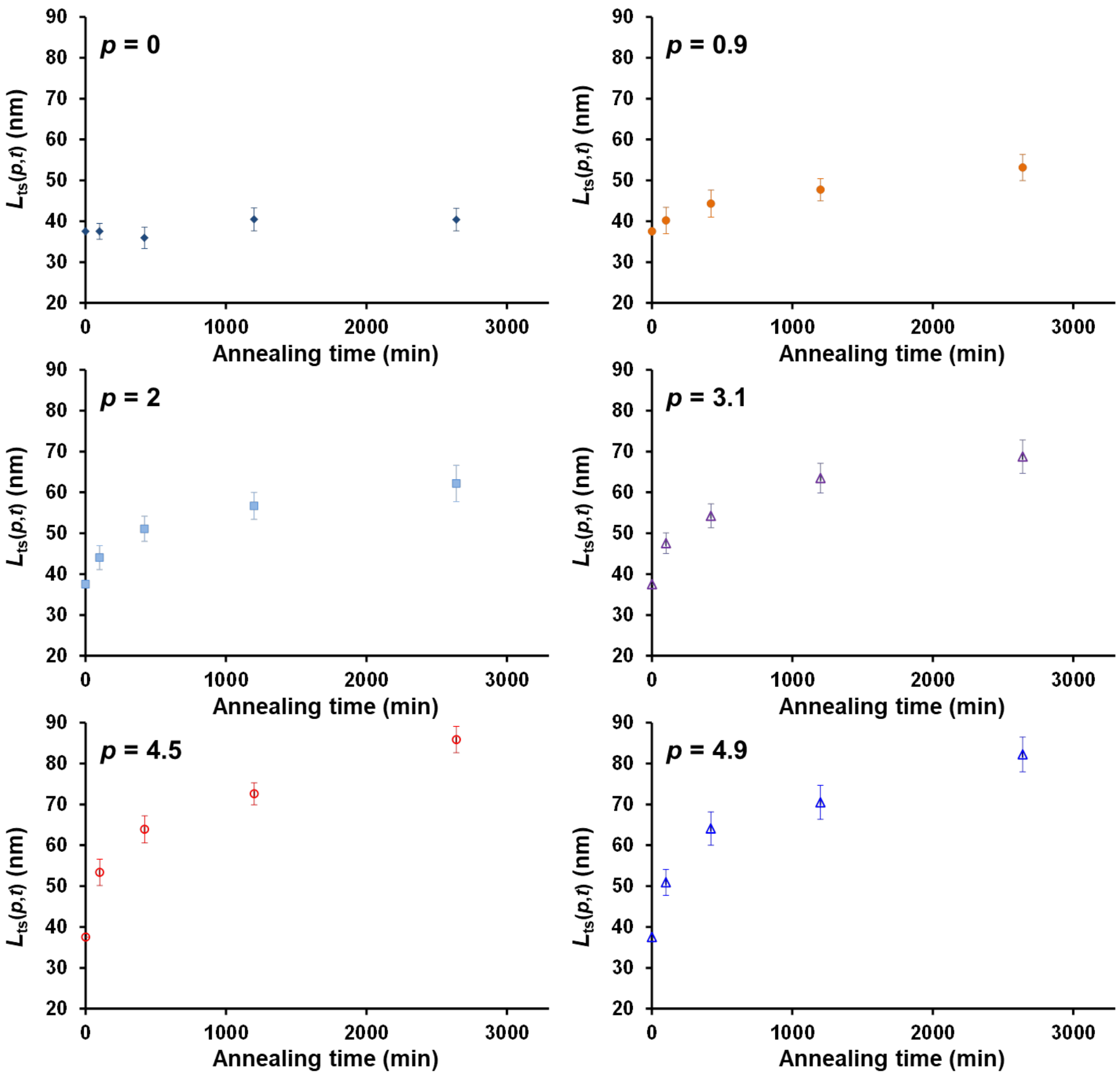

Figure S17. Evolution of the number average lengths of the number average lengths of trapped seeds as a function of time for *p* = 0, 0.9, 2, 3.1, 4.5 and 4.9. *p* is the ratio of the mass of unimer added to the hot seed solution, $m_{uni,added}$, to the mass of seeds originally present in the solution, $m_{seeds,RT}$. Error bars correspond to the SEM of the length distributions.

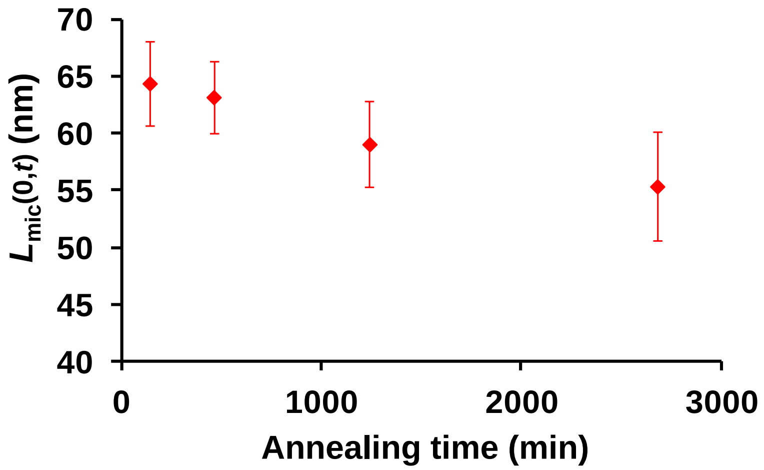

Figure S18: Evolution of the number average lengths of PFS_{53}-*b*-PI_{637} seed crystallites annealed in decane at 75 °C, and cooled to 23 °C, as a function of the annealing time (control experiment).

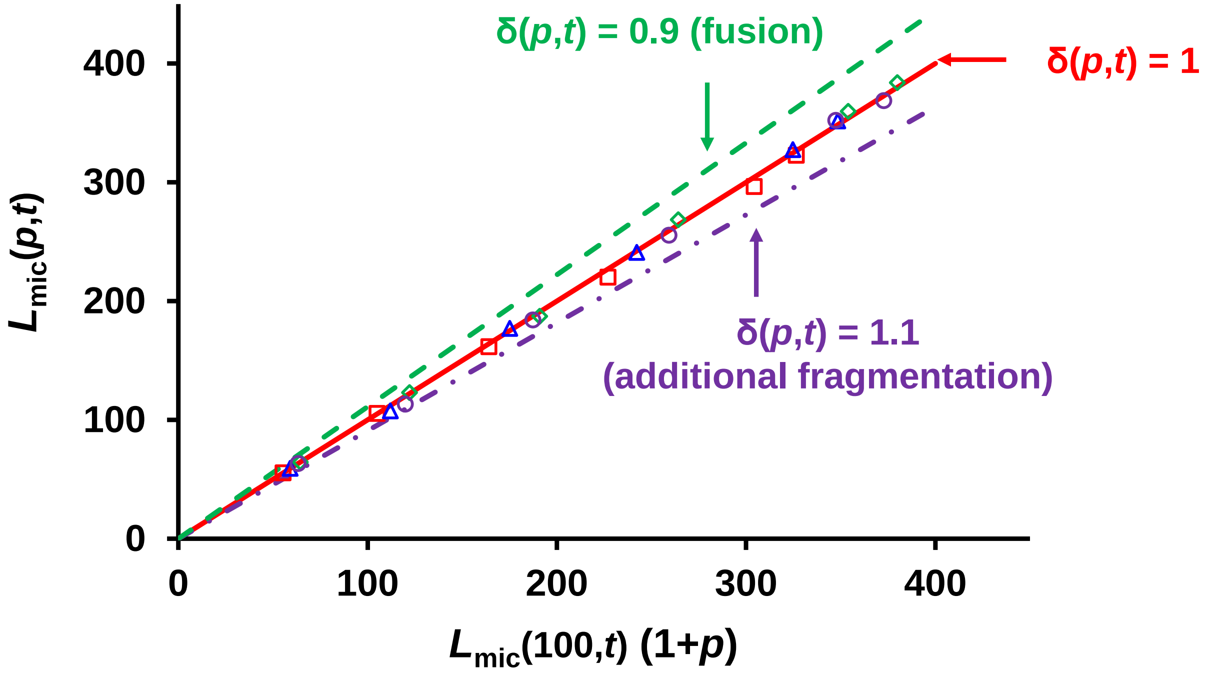

Figure S19: Plot of $L_{mic}(p,t)$ as a function of (1+p) $L_{mic}(100,t)$ (symbols). The three lines correspond to three different scenarios: Addition of PFS_{53}-*b*-PI_{637} unimer induces fragmentation ($\delta(p,t)$ =1.1, purple dot-dashed line), promotes fusion between seeds ($\delta(p,t)$ = 0.9, green dashed line), or does not affect the number of seeds ($\delta(p,t)$ = 1, red line).

References

1. Guerin, G.; Rupar, P.; Molev, G.; Manners, I.; Jinnai, H.; Winnik, M.A. Lateral Growth of 1D Core-Crystalline Micelles upon Annealing in Solution. *Macromolecules* **2016**, *49*, 7004–7014, doi:10.1021/acs.macromol.6b01487.
2. Guerin, G.; Molev, G.; Rupar, P.A.; Manners, I.; Winnik, M.A. Understanding the Dissolution and Regrowth of Core-Crystalline Block Copolymer Micelles: A Scaling Approach. *Macromolecules* **2020**, *53*, 10198–10211, doi:10.1021/acs.macromol.0c02215.
3. Zhou, Y.; Hu, W. Kinetic Analysis of Quasi-One-Dimensional Growth of Polymer Lamellar Crystals in Dilute Solutions. *J. Phys. Chem. B* **2013**, *117*, 3047–3053, doi:10.1021/jp311889n.
4. Boott, C.E.; Leitao, E.M.; Hayward, D.W.; Laine, R.F.; Mahou, P.; Guerin, G.; Winnik, M.A.; Richardson, R.M.; Kaminski, C.F.; Whittell, G.R.; et al. Probing the Growth Kinetics for the Formation of Uniform 1D Block Copolymer Nanoparticles by Living Crystallization-Driven Self-Assembly. *ACS Nano* **2018**, *12*, 8920–8933, doi:10.1021/acsnano.8b01353.
5. Johnston, D.C. Stretched Exponential Relaxation Arising from a Continuous Sum of Exponential Decays. *Phys. Rev. B* **2006**, *74*, doi:10.1103/PhysRevB.74.184430.
